# Supplementary material for: Single particles as resonators for thermomechanical analysis
Source: Nat Commun. 2020 Mar 6;11:1235. doi: 10.1038/s41467-020-15028-y (PMC7060253; doi:10.1038/s41467-020-15028-y)
Supplement: Supplementary file 1 — Supplementary Information [file 41467_2020_15028_MOESM1_ESM.pdf]

Supplementary Information

Single Particles as Resonators for Thermomechanical Analysis

Okeyo, Peter Ouma, et al.

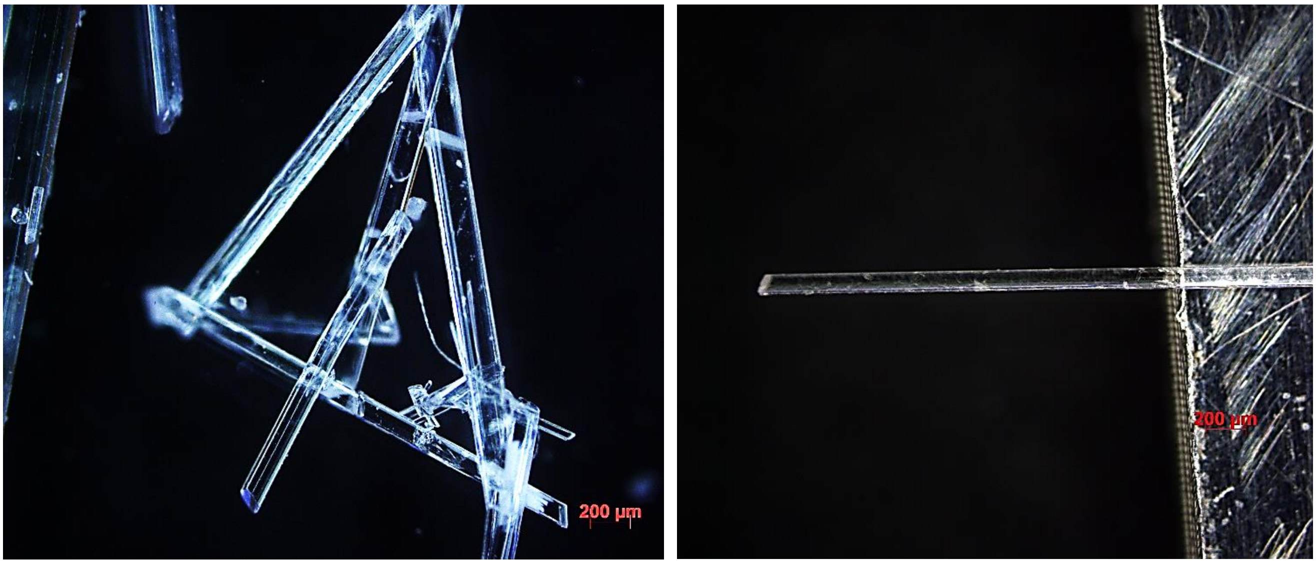

Supplementary Figure 1. Heterogeneous single particles. Optical images of single particles of theophylline monohydrate (TP MH) showing the defects that can be present on the surface of the particle.

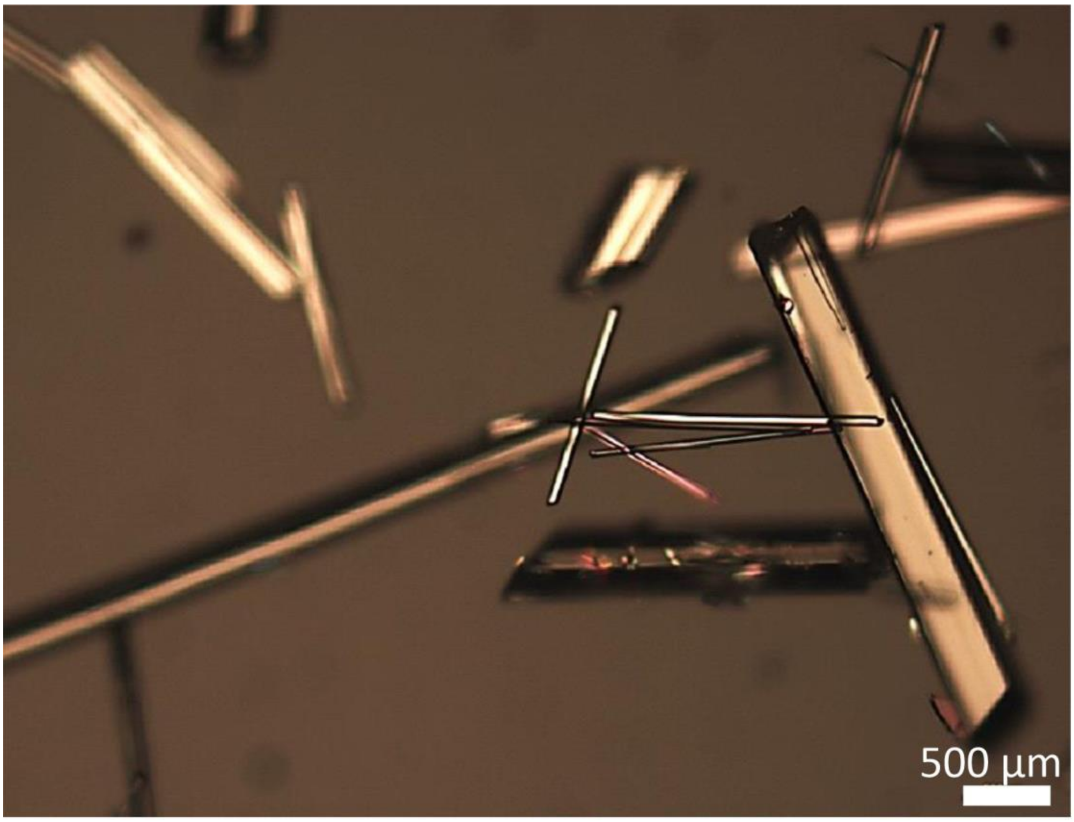

Supplementary Figure 2. Different shapes of TP MH particles. Multiple particles of TP MH that were obtained via slow evaporative recrystallization. This optical image shows different shapes (plate, rod like) of TP MH particles that were studied.

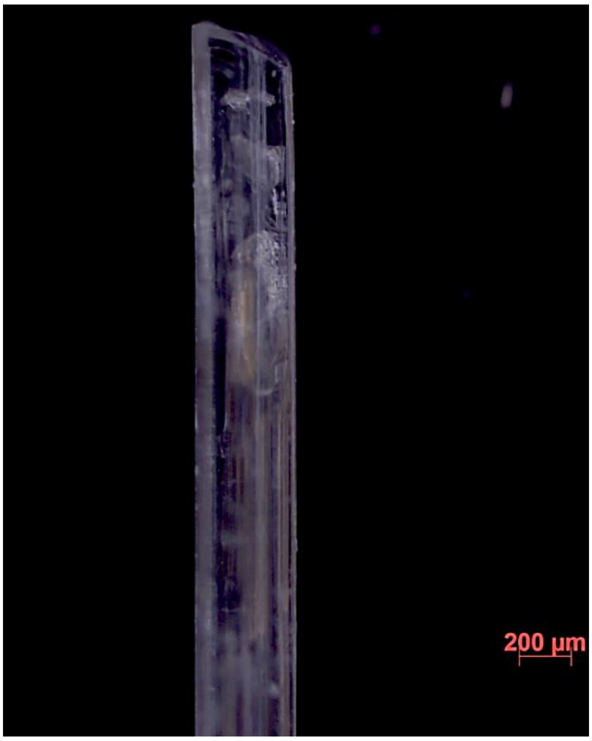

Supplementary Figure 3. TP MH cantilever. Optical image of a plate structure of a TP MH particle that was treated as a cantilever.

Supplementary Note 1 Particle analysis

Optical images of different structures of These images are showing the heterogeneity of the particles that were used in the study as well as the different shapes that can arise from this synthetic steps. TP MH particles were recrystallized via slow evaporative recrystallization. The advantage of obtain such particles are that they vary in their thermomechanical response and such variations can be detected with the proposed PMTA method. The challenge here with the standard bulk methods is that such differences are not only challenging to decipher, but they can also be undetectable as demonstrated with the examples in the main text.

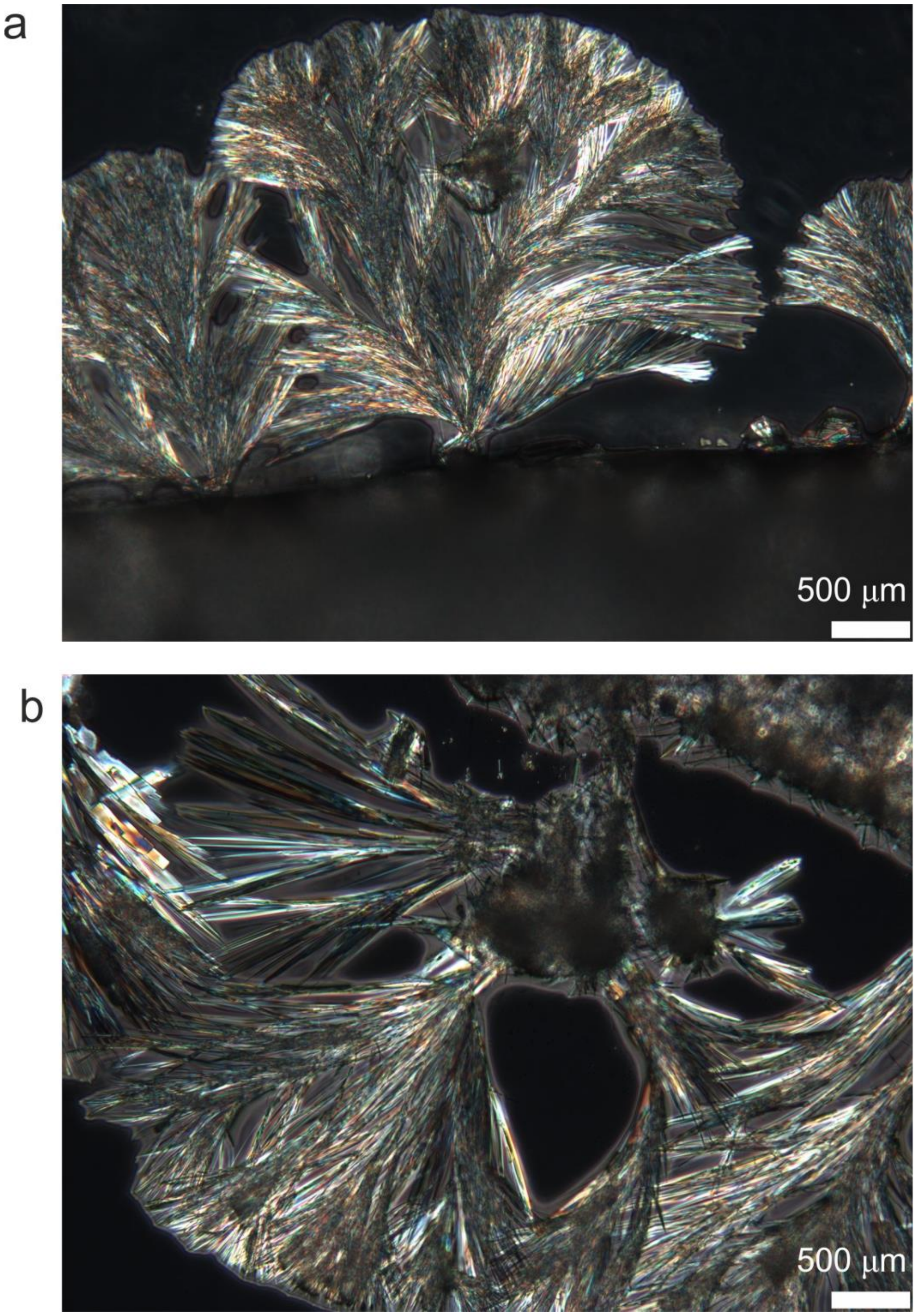

Supplementary Figure 4. Recrystallized particles (evaporative crystallization). Polarised Light microscopy (PLM) images of clustered particles of TP MH after recrystallization with different shapes and sizes as well as visible defects.

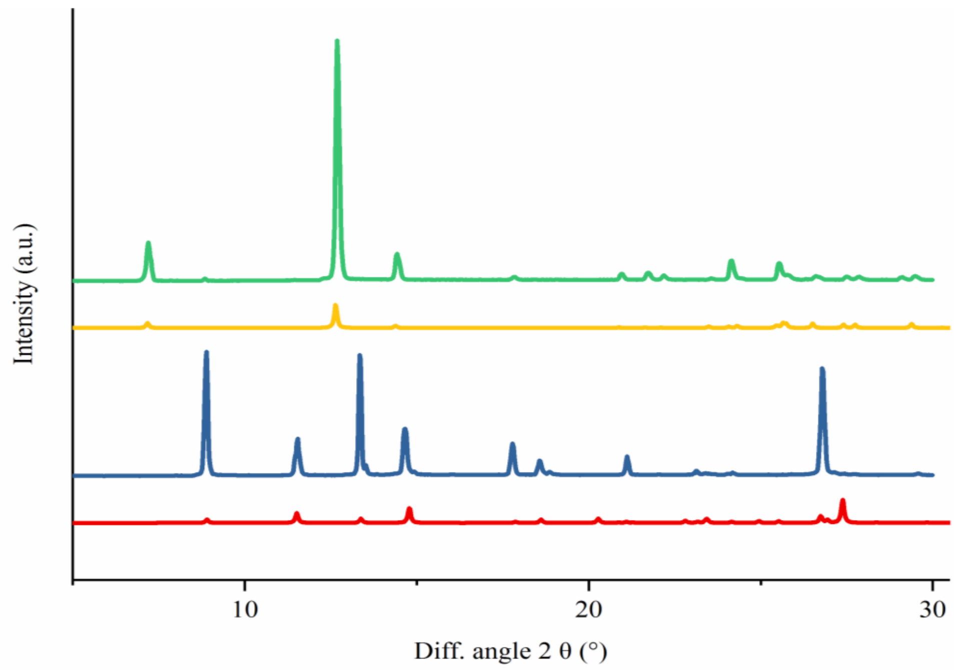

Supplementary Figure 5. XRPD diffractograms of TP. Experimental and calculated (Cambridge Structural Database) XRPD diffractograms of TP AH form II (green), CSD ref code: BAPLOT01 (yellow), and **b)** TP MH (blue), CSD ref code: THEOPH01(red).

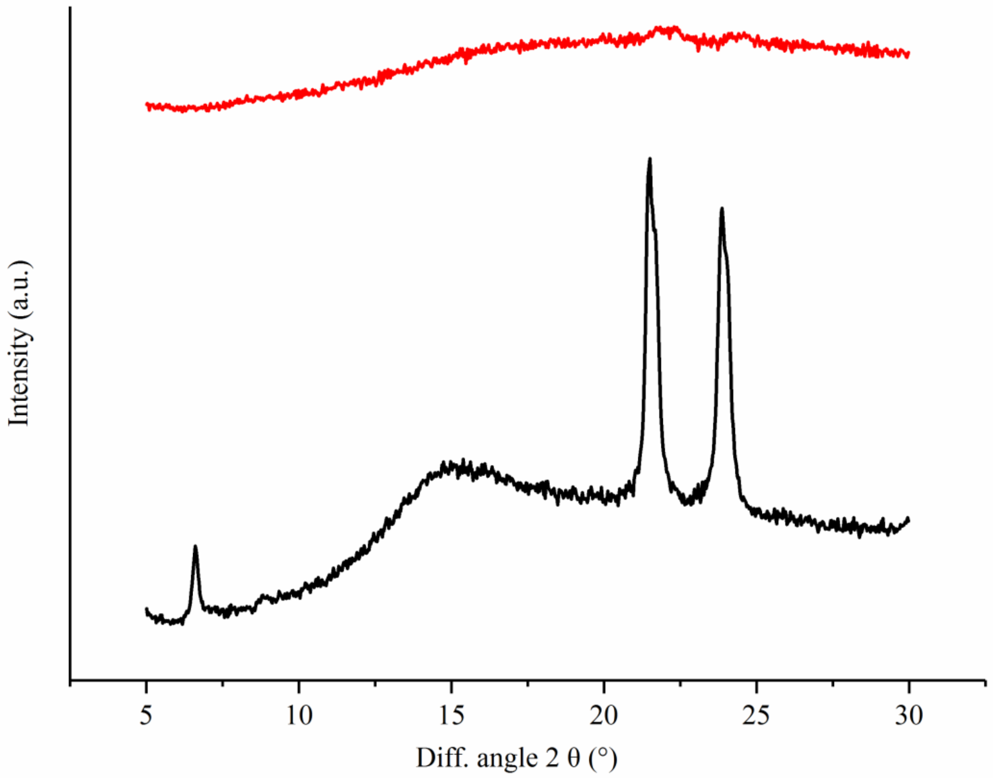

Supplementary Figure 6. Comparison between hydrated and anhydrous collagen samples. XRPD diffactograms of fibres type I hydrated (black) and dry (red).

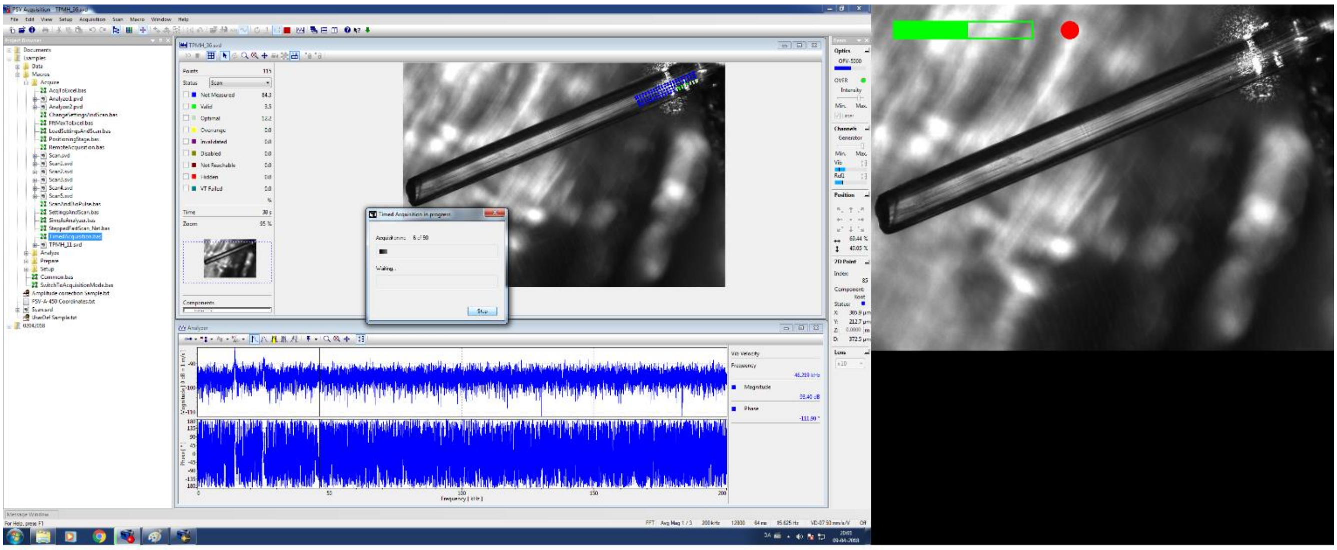

Supplementary Figure 7. Example of a scanning grid on a TP MH particle. Visualisation of a scanning grid (blue points on the particle) using the PSV software provided from Laser Doppler Vibrometer (LDV) on a TP MH particle during a measurement run at room temperature. The green bar at the top of the figure is the signal strength of the reflected beam from the laser on the particular point being measured.

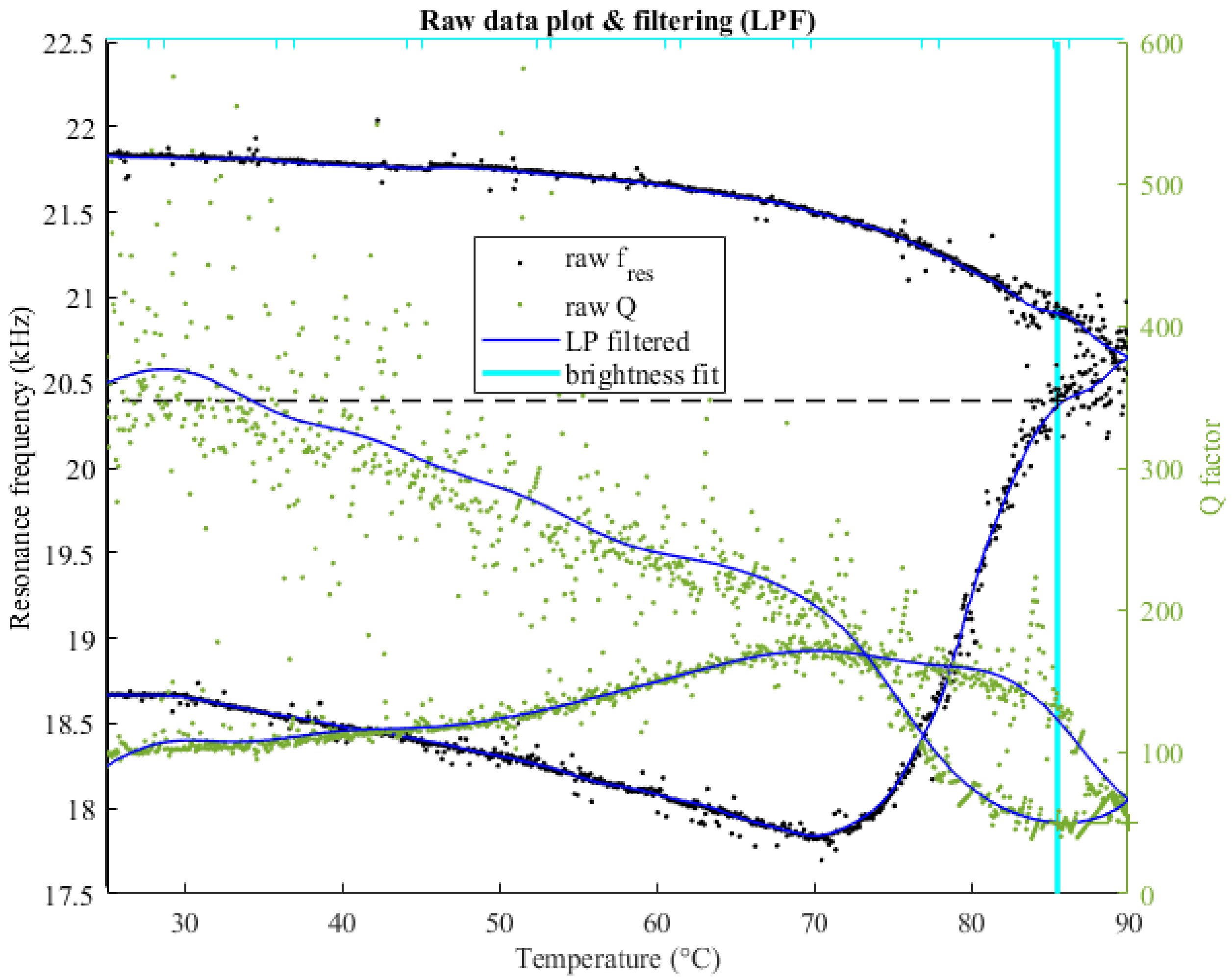

Supplementary Figure 8. PMTA thermogram of TP MH. Raw & filtered data for the tracking of resonance frequency and quality factor of TP MH (1000 µm x 65 µm) during heating and cooling (25 – 90-25 °C, 5 °C/min). The turquoise line in this plot marks where the water has been lost in the crystalline hydrate particle.

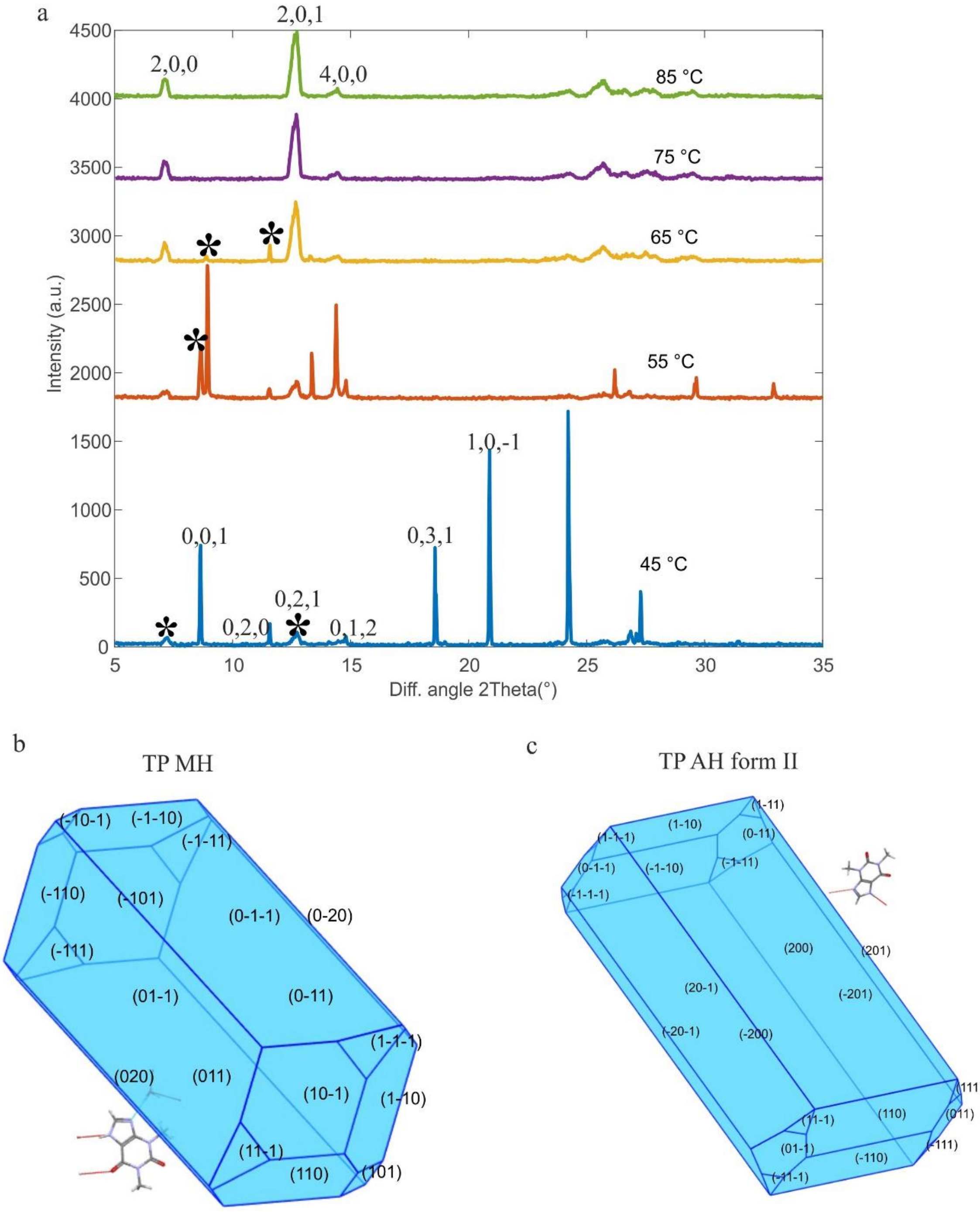

Supplementary Figure 9 a) VT-XRPD dehydration of TP MH (25 – 85 °C, 5 °C/min). VT-XRPD showing the dehydration of TP MH to its anhydrous form where the astrix (*) are showing changes in the unit cell dimensions of TP during this process.The same batch of particles were used for this measurement as in Supplementary Figure 1, **b-c)** Bravais-Friedel-Donnay-Harker (BFDH) was used in order to predict the morphology of TP MH (CSD ref code: THEOPH01) and TP AH (CSD ref code: BAPLOT01).

Supplementary Note 2 Correlations between thermal transitions in TP

The PMTA thermogram in Figure 3b shows that there are thermal transitions occur at 45 °C and 70 °C, which are proposed to be due to the presence of metastable solid forms that appear during the dehydration of TP MH to its stable anhydrous form. By performing VT-XRPD, it can be seen that there are indeed thermal transitions occurring at this similar temperature that are linked to changes in the unit cell dimensions in particular its contraction as can visually be observed from the two morphology predictions of TP MH and TP AH form II. In addition to this dehydration studies were also performed using Raman line-focus microscopy on TP MH. The TP MH particles that were used in our previous Raman paper were also used to perform the experiments in this manuscript. In the Raman paper it was found that different metastable forms of TP could be visualised on different regions of a single particle thereby highlighting the heterogeneity associated with these single particles during dehydration. The temperature ramping experiments were performed and showed thermal transitions at 45 and 70 °C that coincide with the presence of metastable forms of TP. The validation of these metastable forms using three different analytical techniques gives a strong case that what the PMTA is detecting during these subtle thermal transitions are metastable forms.

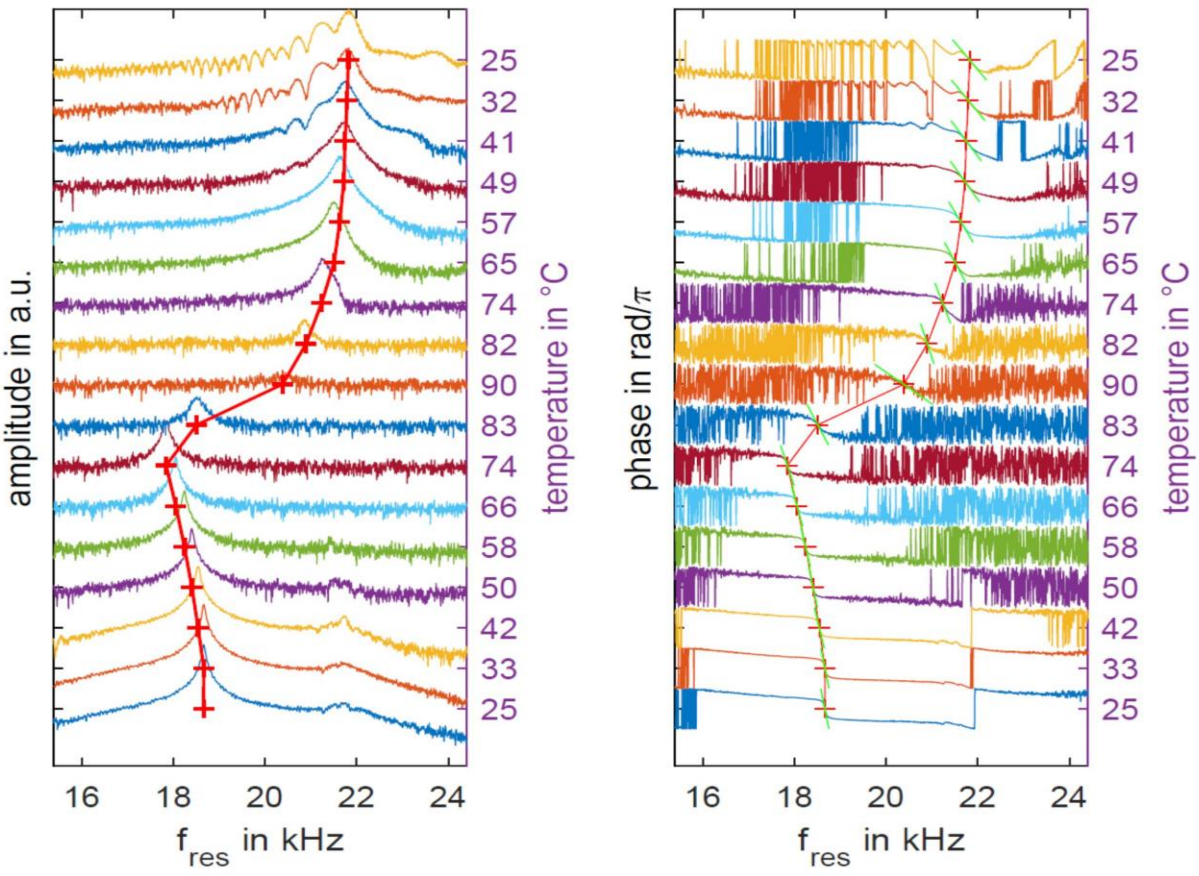

Supplementary Figure 10. Manual tracking of f_{res} and Q factor. Raw thermomechanical spectra and the phase response (Q factor) of TP MH (25-90°C, 5 °C/min)

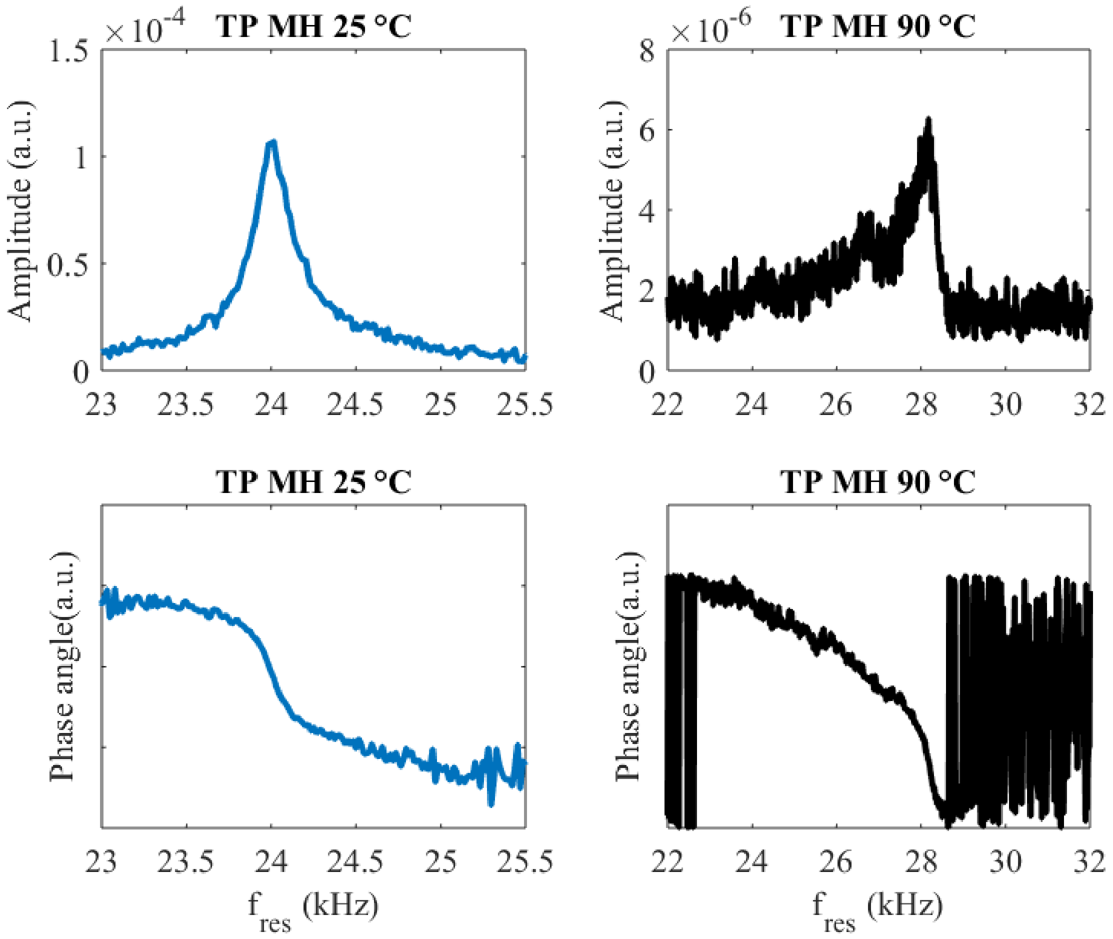

Supplementary Figure 11. **Thermomechanical spectra and phase response of TP MH particle.**

It was observed that during the experiments there would be a change in the raw spectra and phase response as highlight by the raw spectra in the figure above. These changes are attributed as discussed in the manuscript to the formation of different phases in particular hydrate to anhydrous particle, which have different physical and mechanical properties that result in changes in the spectra and phase.

Lumped Element Model

Real life systems are much more complex than the idealised systems that are presented by the Euler Bernouilli beam theory and this is even more complex with the heterogeneous particles that were used in our studies as a result the lumped element model was used to help explain the dampening of the particles.

As shown Equation 1 in the main text was used in order to track the slope of Q and the Supplementary Equation 1 was used in order to describe the factors that were considered for the particle resonators that were used in this this study.

$$\frac{1}{Q} = \frac{1}{Q_{mat}} + \frac{1}{Q_{air}} + \frac{1}{Q_{surr}} + \ldots \quad (1)$$

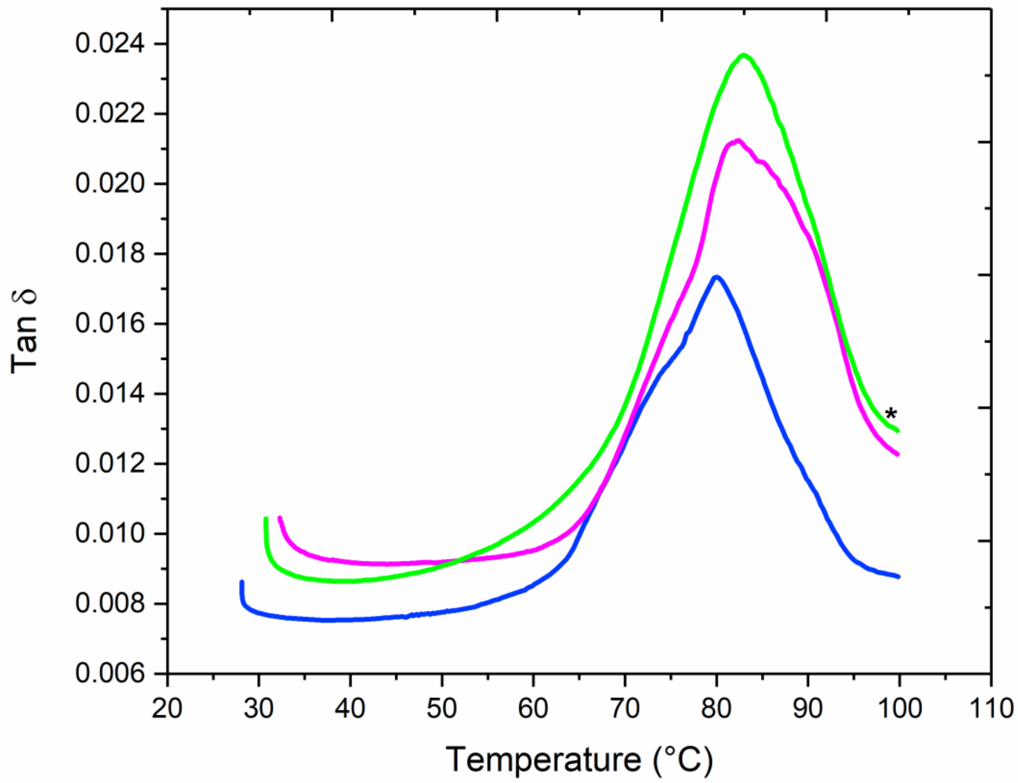

Supplementary Figure 12. Triplicate DMA thermograms of TP MH (tan δ). Tan δ signals showing major transition temperature determined from samples containing TP MH. *indicate signal obtained when impurity (caffeine, anhydrous TP) was added to TP MH sample.

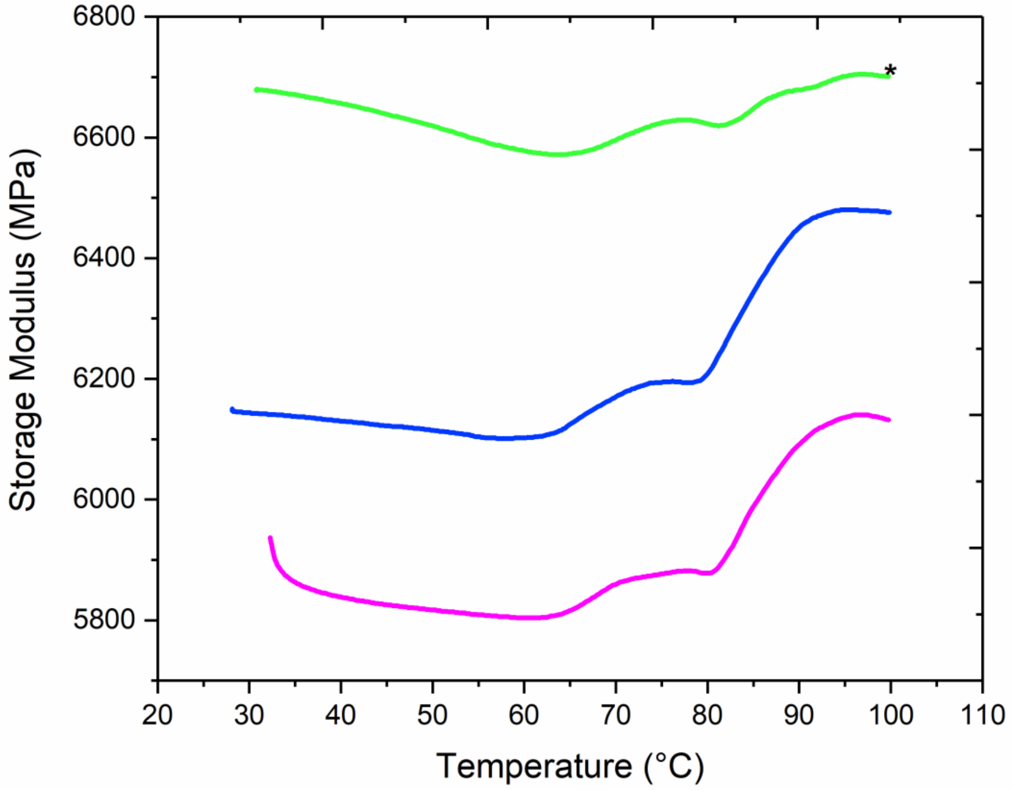

Supplementary Figure 13. Triplicate DMA thermograms of TP MH (storage modulus). Storage modulus signals showing transition temperature determined from samples containing TP MH.

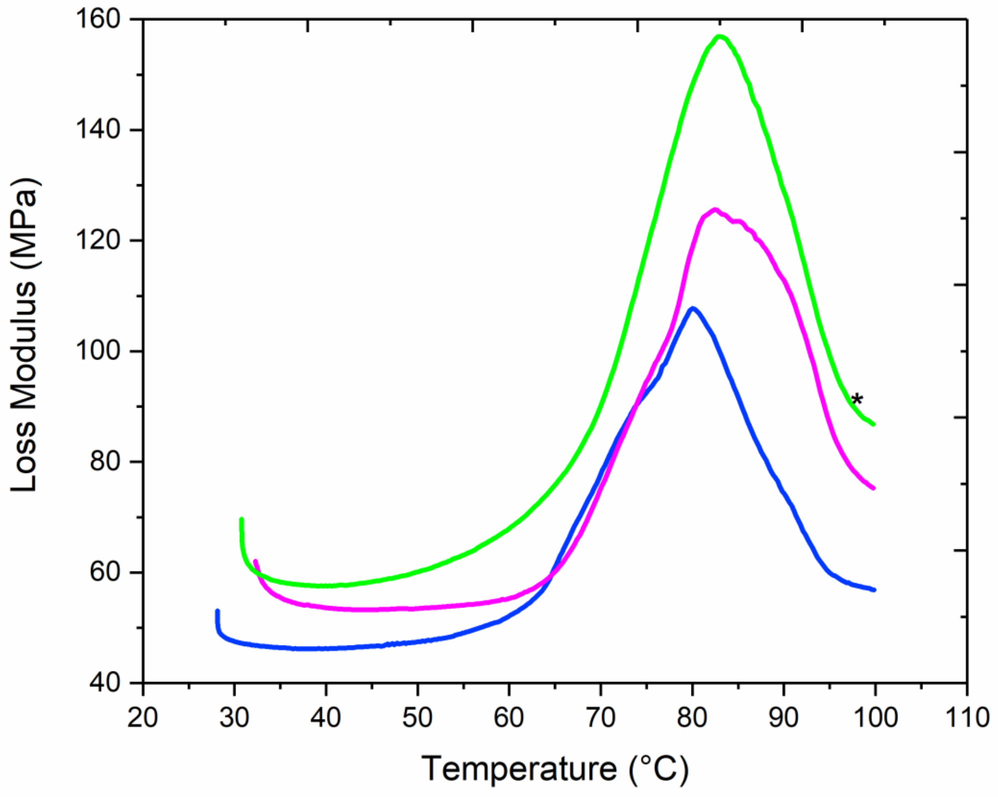

Supplementary Figure 14. Triplicate DMA thermograms of TP MH (loss modulus). Loss modulus signals showing transition temperature determined from samples containing TP MH.

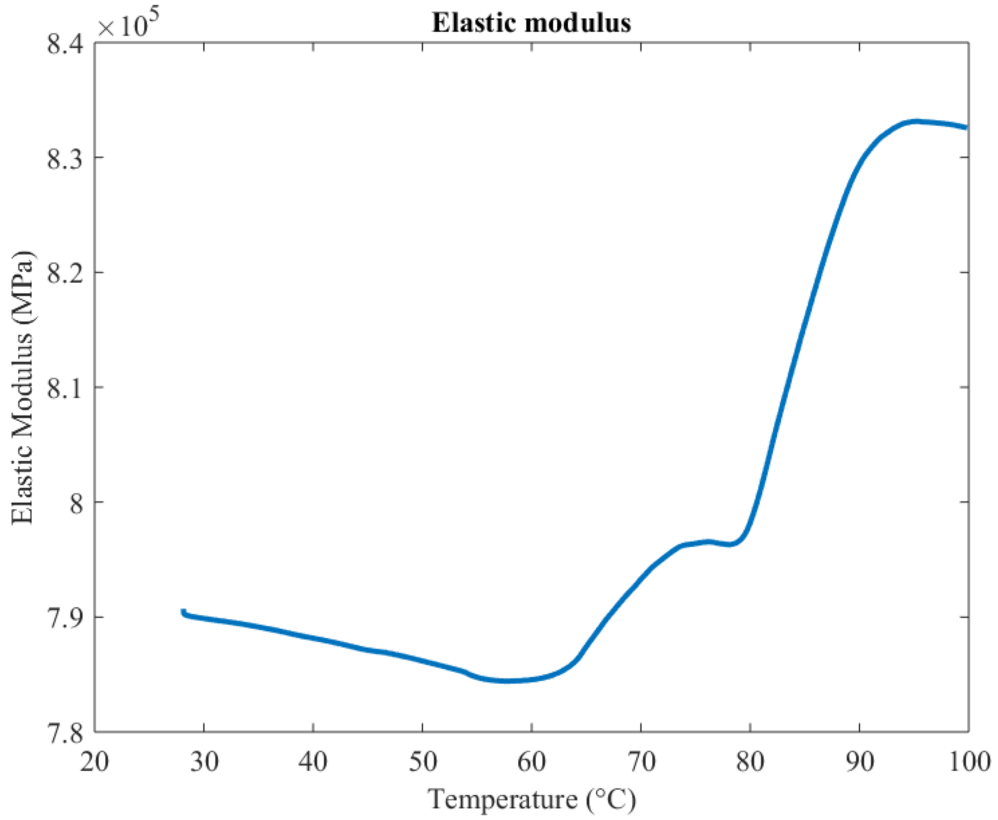

Supplementary Figure 15. DMA thermogram of TP MH during dehydration (25 - 100 °C, 5 °C/min).

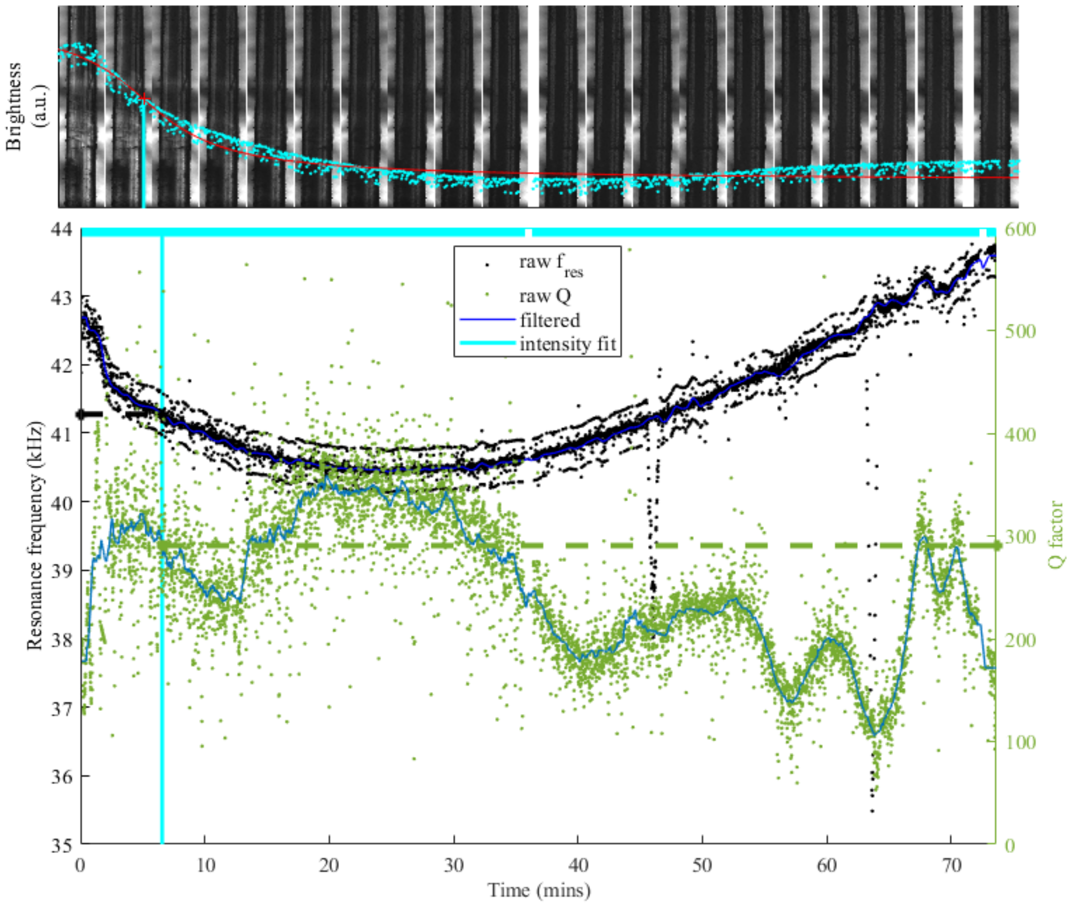

Supplementary Figure 16. a) Raw data showing TP MH dehydration at 50 °C for 90 minutes. Optical images showing the dehydration of TP MH at 50 °C Raw showing the tracking of the resonance frequency and Q factor during the isothermal dehydration of an individual particle of TP MH.

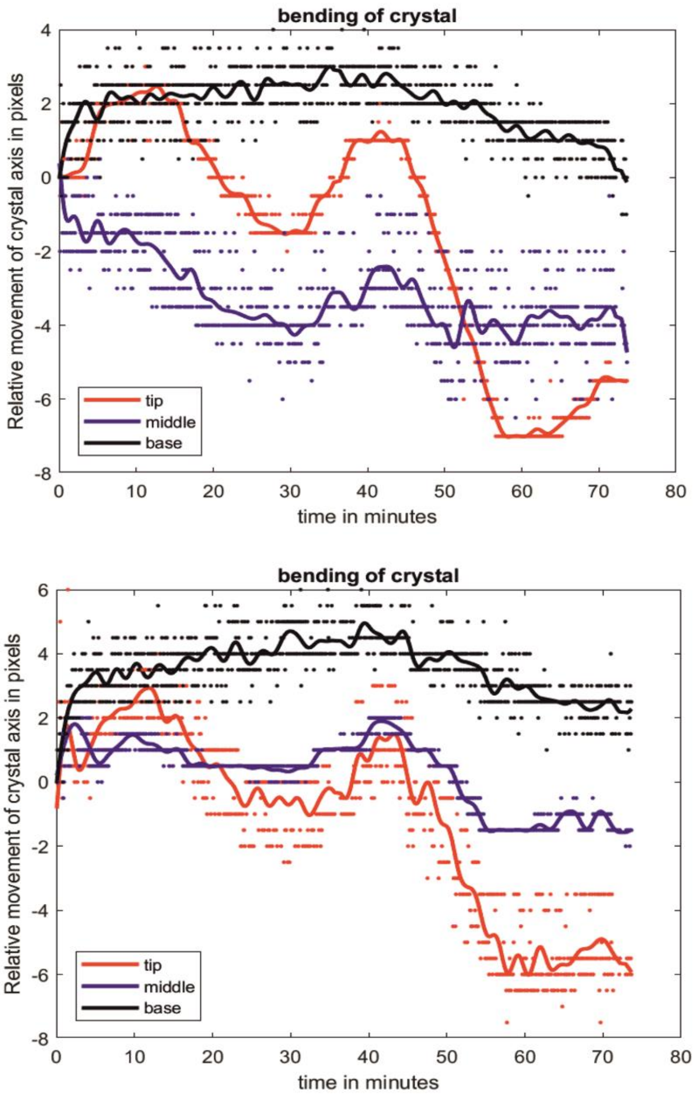

Supplementary Figure 17. Bending of a TP MH cantilever particle during an isothermal measurement.

The bending of the particle was more pronounced at the tip in comparison to the base and the middle of the particle. The movement at the base of the particle was less in comparison to the middle and ends of the particle. The first two minutes showed that there is a dramatic movement at the tip and this was also observed at the middle of the particle, however, this was not as pronounced as at the end of the particle. This analysis was done twice for reproducibility purposes.

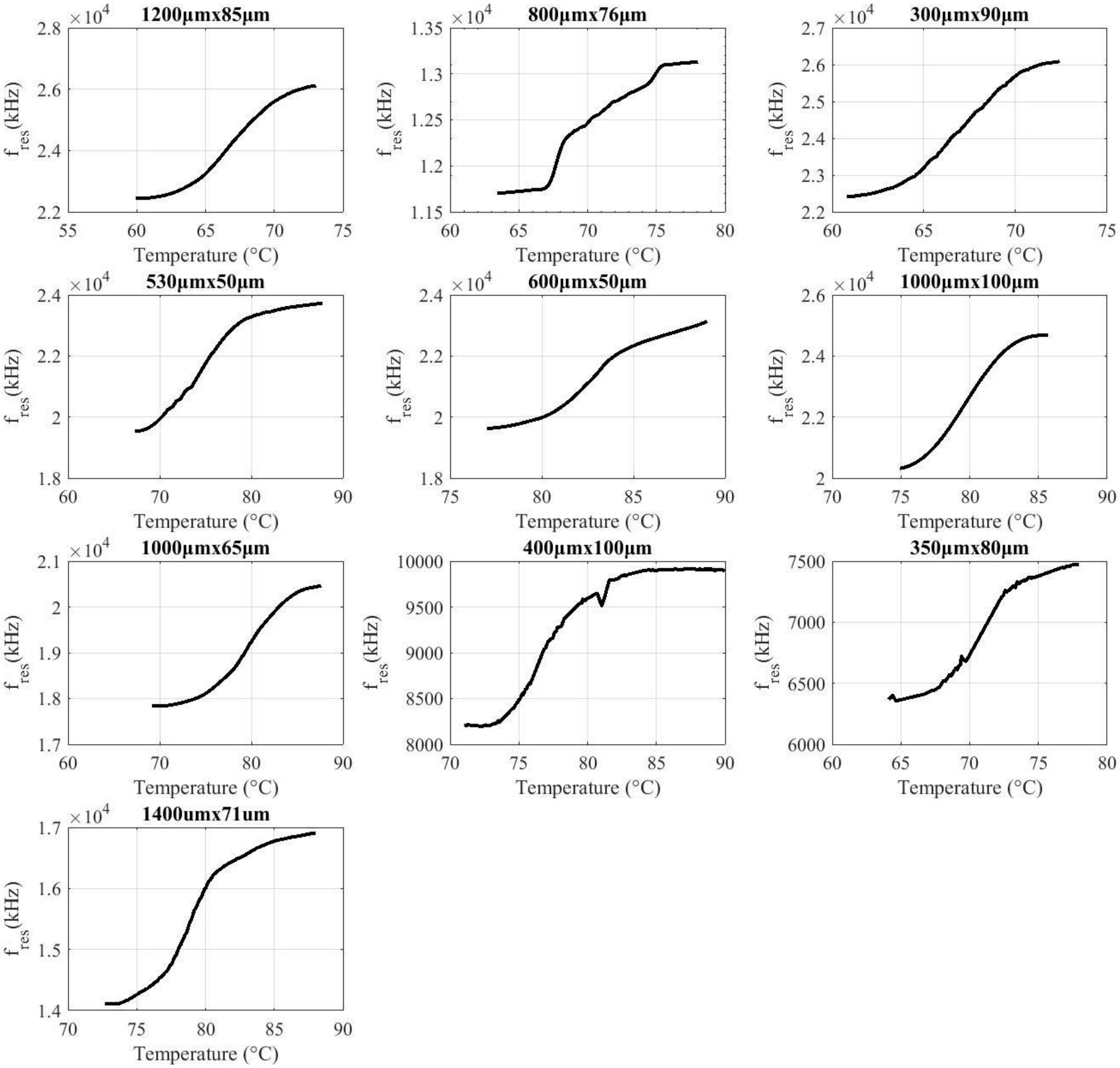

Supplementary Figure 18. Variation associated with TP MH dehydration. Subplots showing the regions that were chosen in the main dehydration event of the TP MH resonators in order to calculate their respective standard deviations.

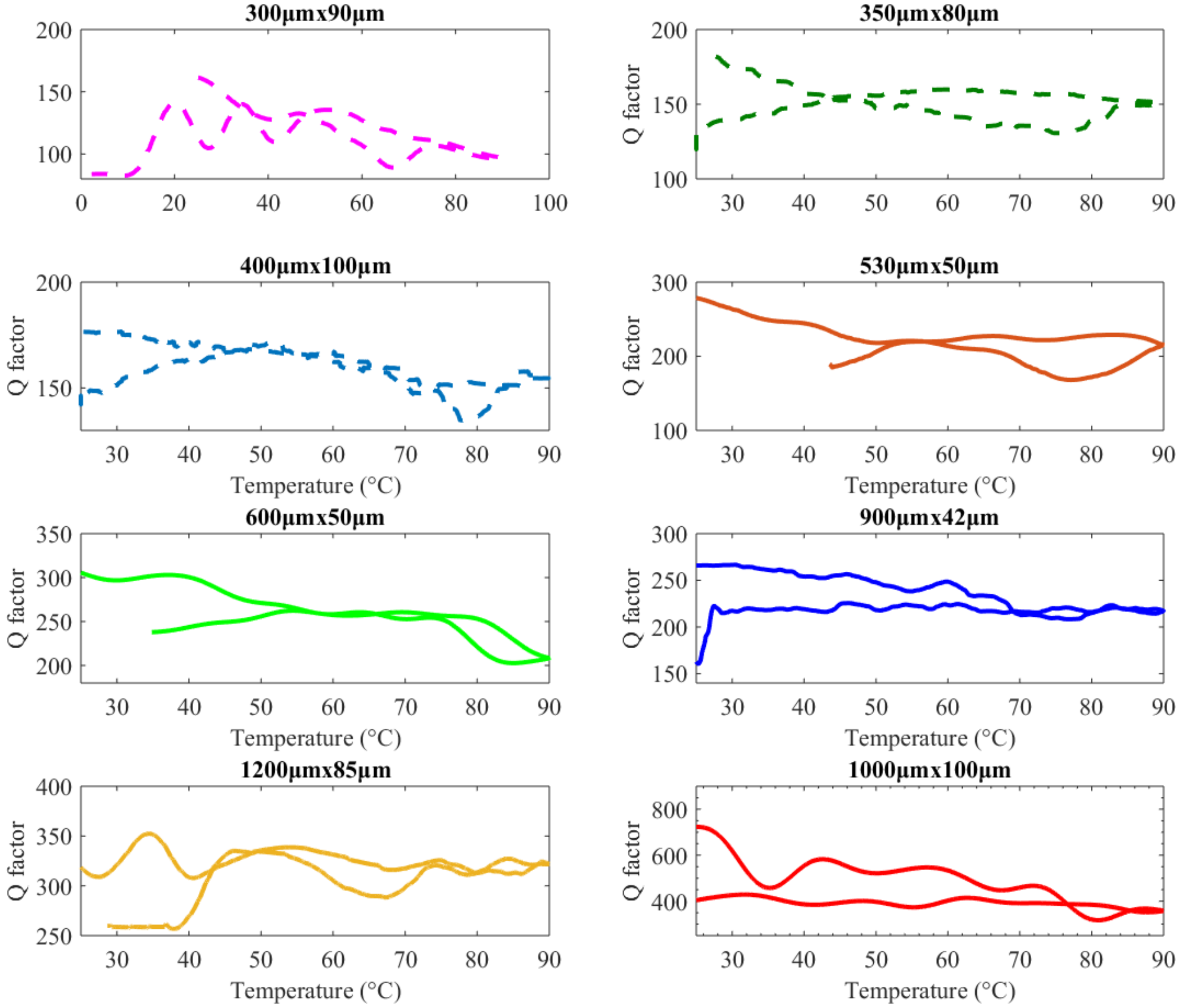

Supplementary Figure 19. Qs for the TP MH resonators in Figure 4. Those not present here can be found in Figures 2, 3, and 5 of the main text

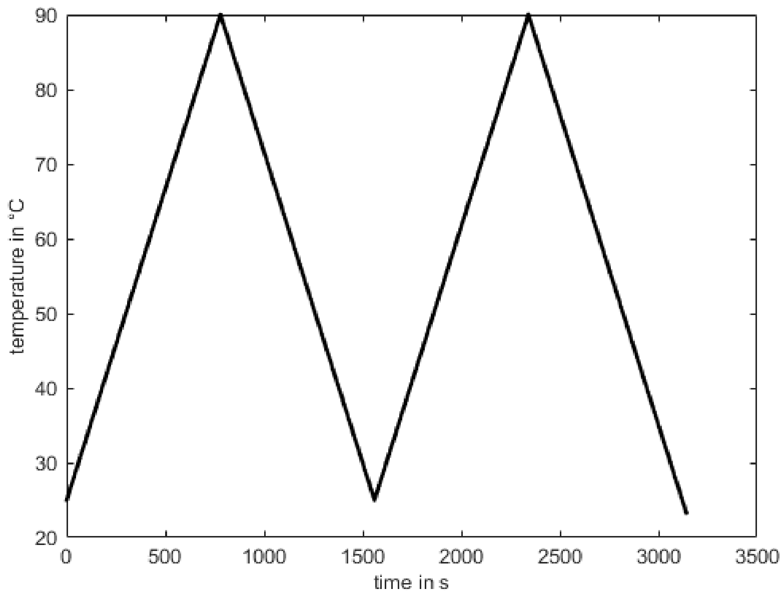

Supplementary Figure 20. Thermal profile for TP MH PMTA experiment. Two heating and cooling cycles (25-90-25-90-25 °C) of TP MH particle in Figure 5 of the main text. The heating profile was obtained from a MATLAB script. The temperature and or time profile of the experiment needs to be done manually via a pop up window prior to extracting the raw data.

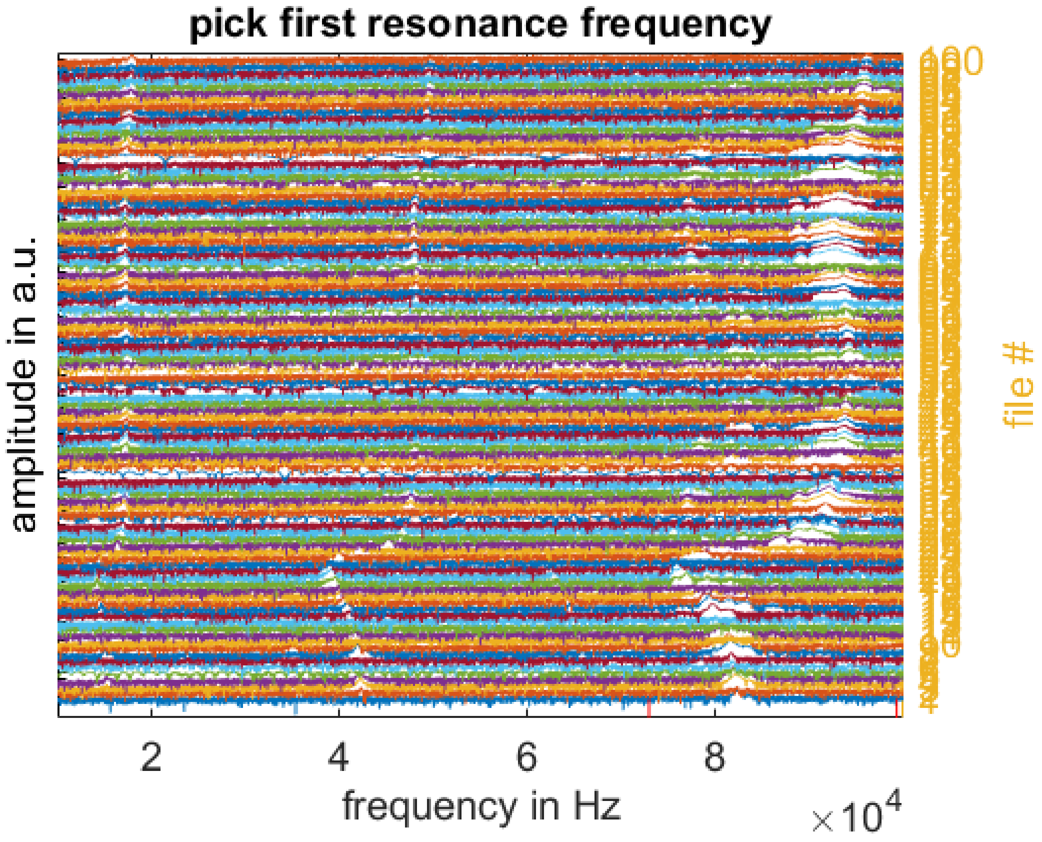

Supplementary Figure 21. Raw thermomechanical spectra. TP MH f_{res} showing notable changes during two heating and cooling cycles (25-90-25-90-25 °C) showing two modes with a relatively good signal to noise ratio and one that is very close to the noise level below 20 kHz. Such modes are challenging to manually track.

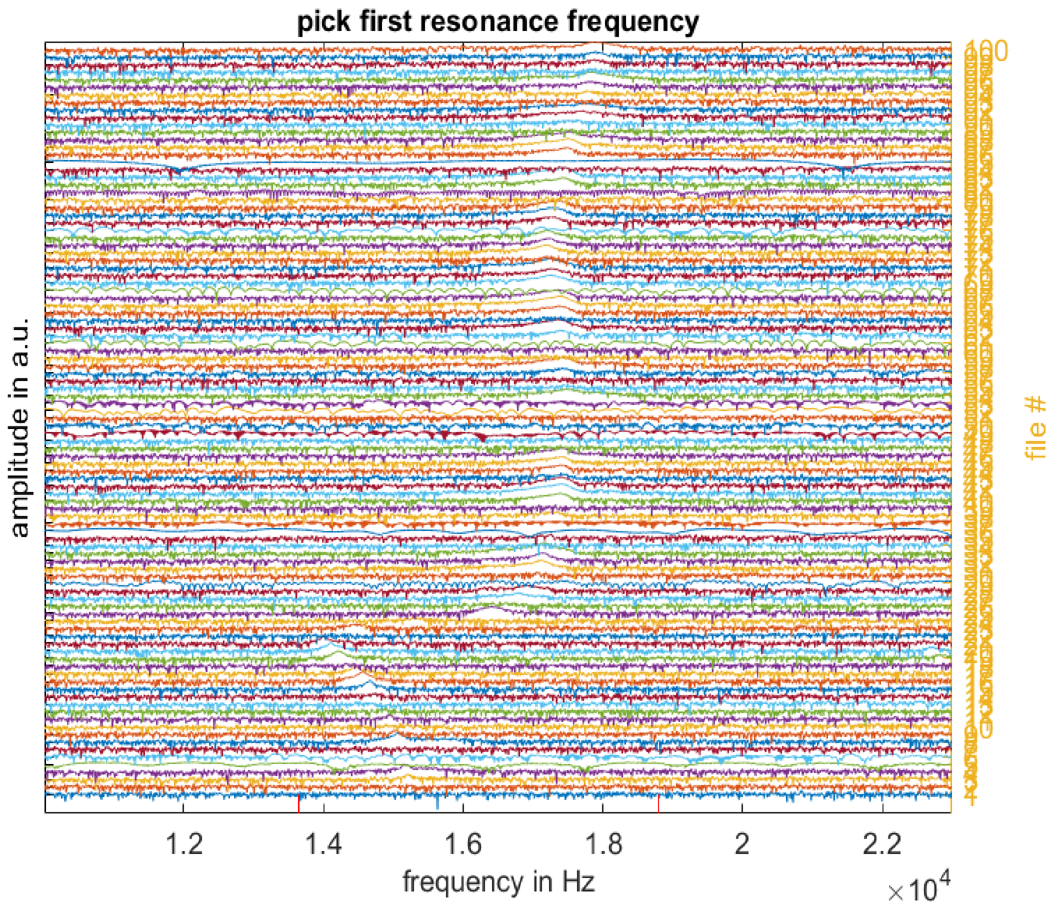

Supplementary Figure 22. Raw thermomechanical spectra of the first mode from *Supplementary Figure 21*

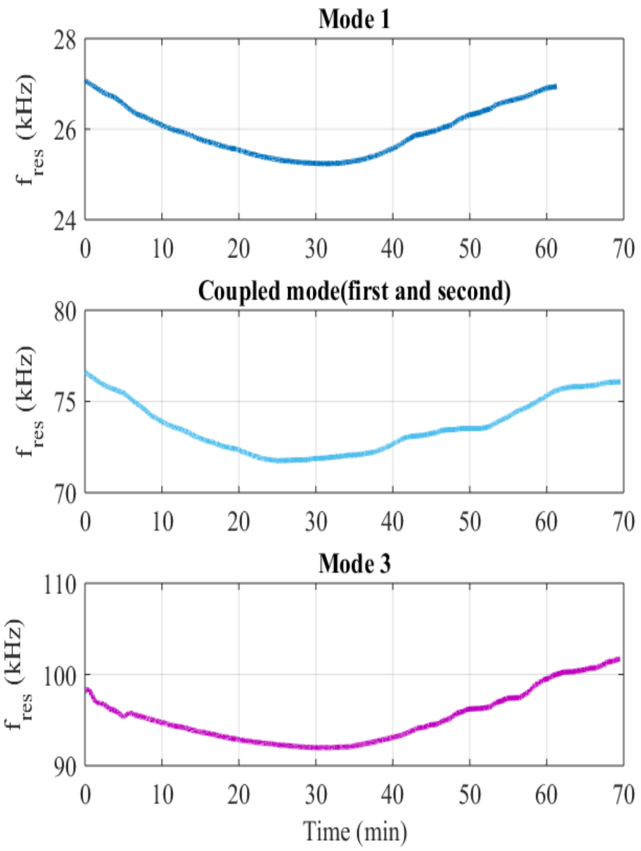

Supplementary Figure 23. Three modes of TP MH particle (1400µm x 74µm) at 50 °C for 70 min.

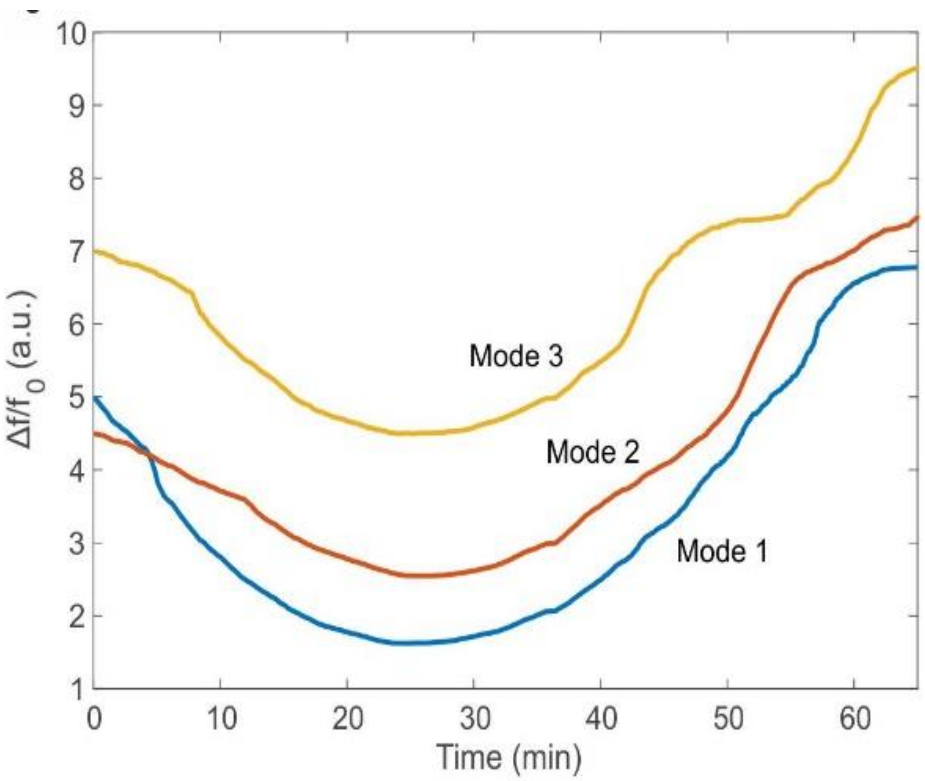

Supplementary Figure 24. Mode analysis. Three flexural modes of vibration from the TP MH particle in Figure 3e (50 °C, 70 min).

These three flexural modes in Supplementary Figure 24 are showing distinct differences in the vibrational modes of TP MH at 50 °C over 70 minutes. Mode 2 shows that there are two distinct thermal transitions that are occurring within the first eleven minutes of the experiment followed by a minor drop in the resonance frequency that recovers after 30 minutes. There is a linear increase from 48 to 53 min then multiple minor thermal transitions. Mode 3 shows the consistent behavior as with mode 2 in the beginning of the experiment, however, the resonance frequency of mode 3 drops at around 9.5 minutes. At 40 min there is a steeper increase in f_{res} in comparison to the other two modes with a short plateau then eventually leads to multiple thermal transitions.

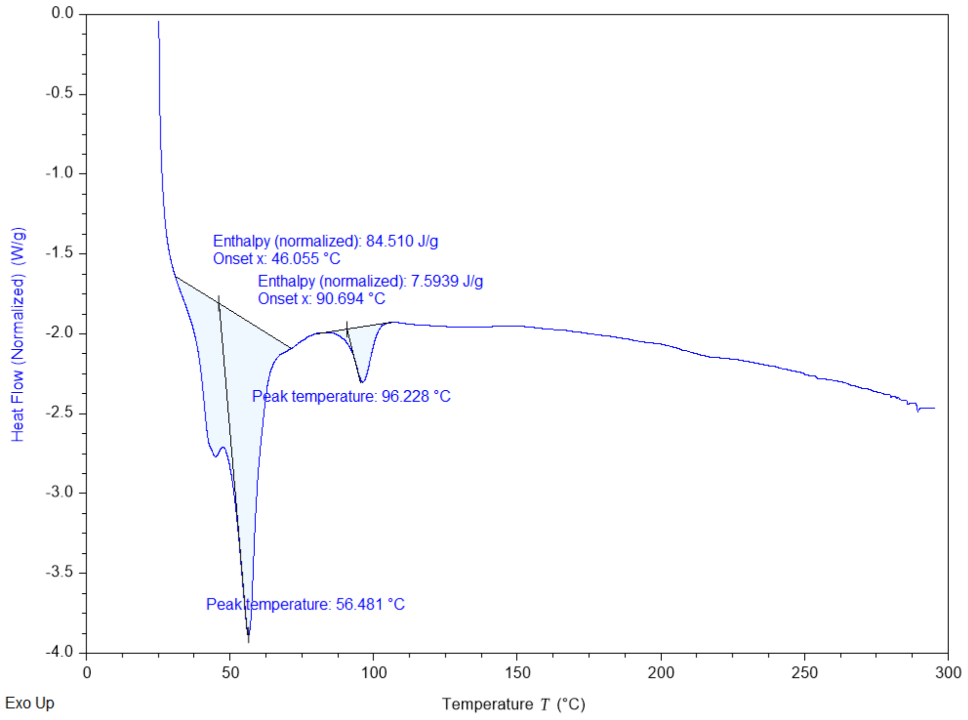

Supplementary Figure 25. DSC thermogram of collagen fibres (25 – 300 °C, 5 °C/ min). A second breakage of the collagen that takes place at 100 °C, which could be associated with water loss

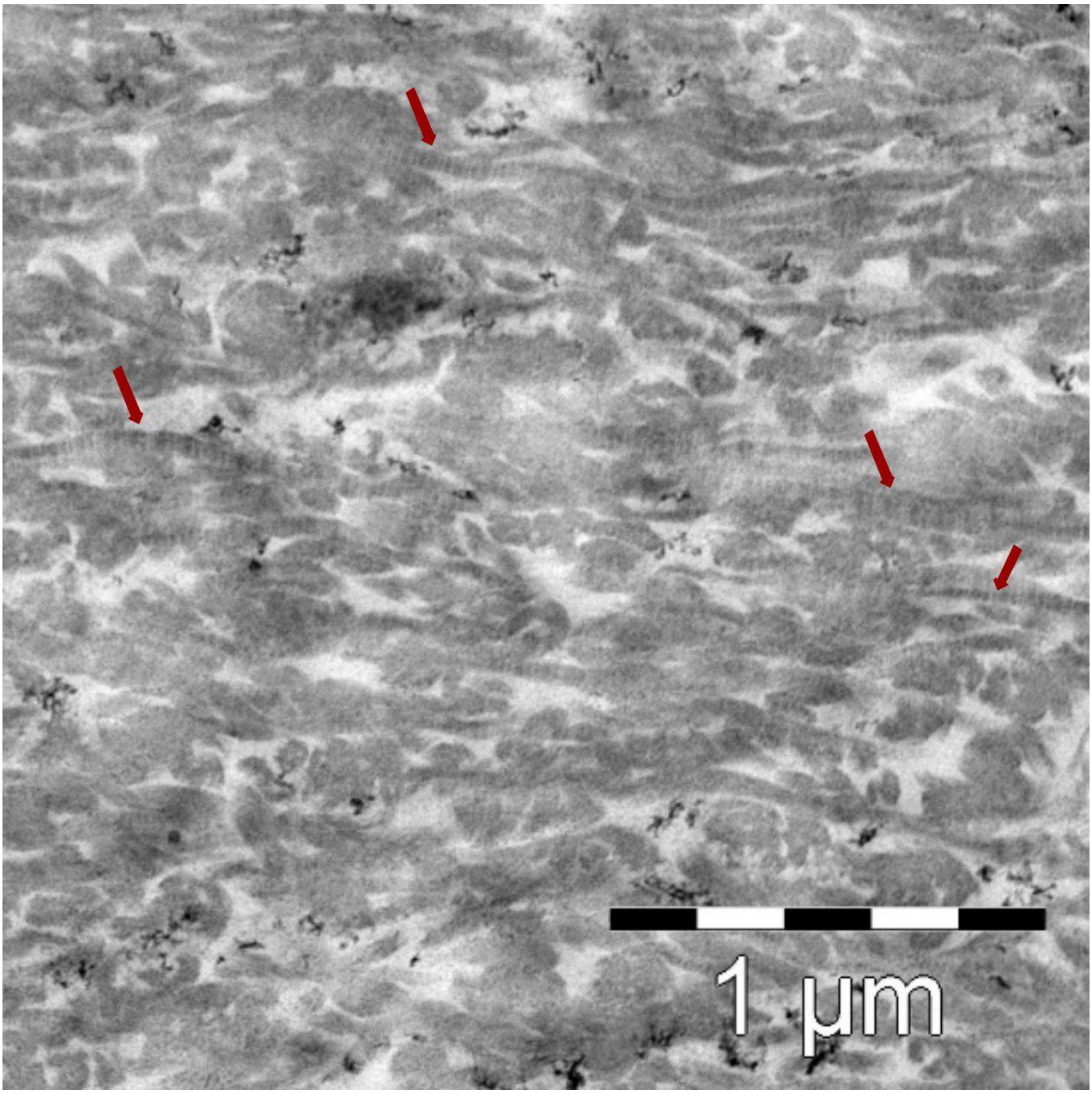

Supplementary Figure 26. Cross section analysis using TEM. Cross section of the fibrous sheet taken for the measurement in Figure 6 of the main text.

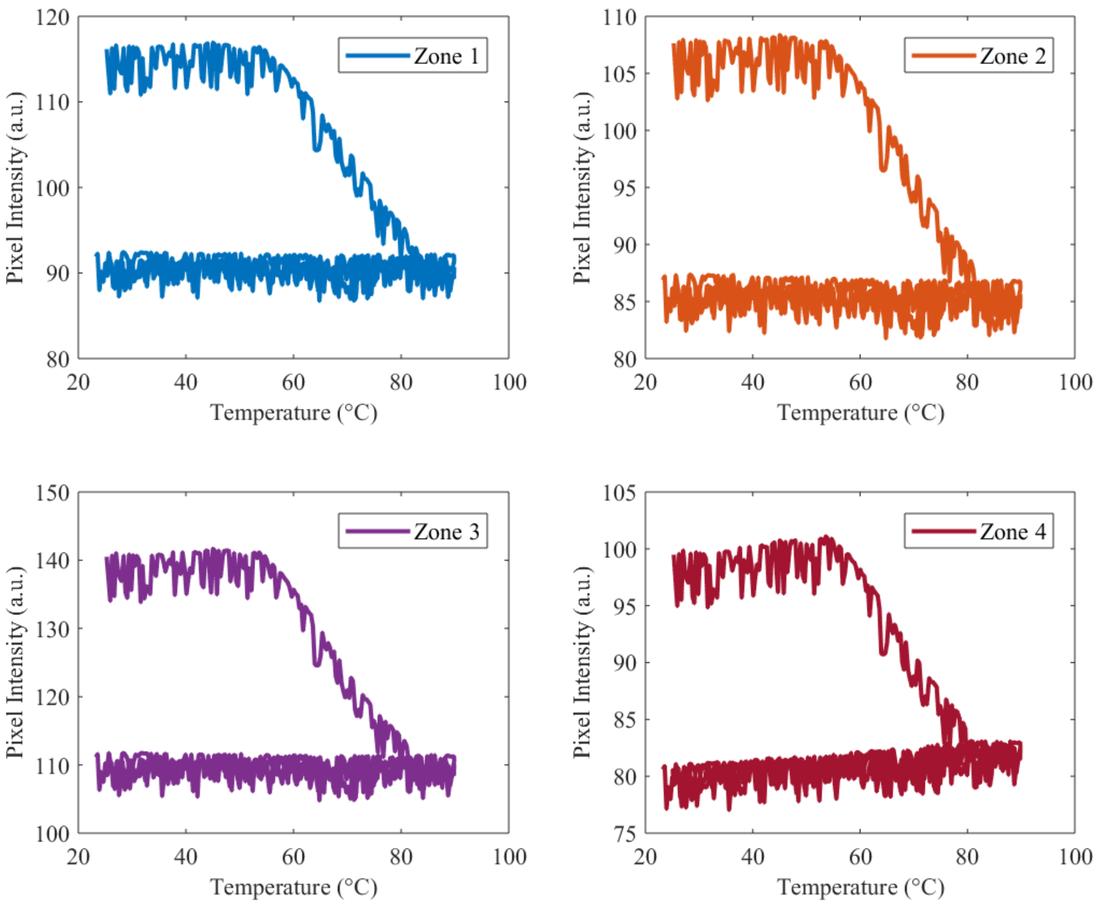

Supplementary Figure 27. Pixel intensity analysis of TP MH during dehydration and cooling. The rough approximation of the pixel intensity in four different zones/regions of a TP MH particle resonator.

FEM Simulations

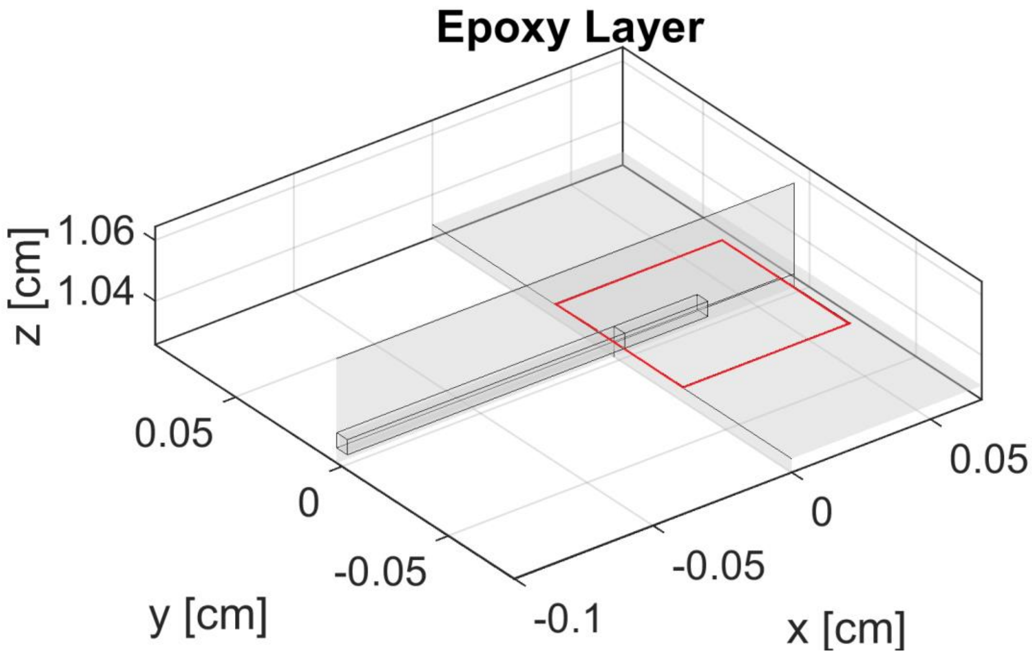

Supplementary Figure 28. Illustration of epoxy glue layer (marked in red) on clamped end of the particle.

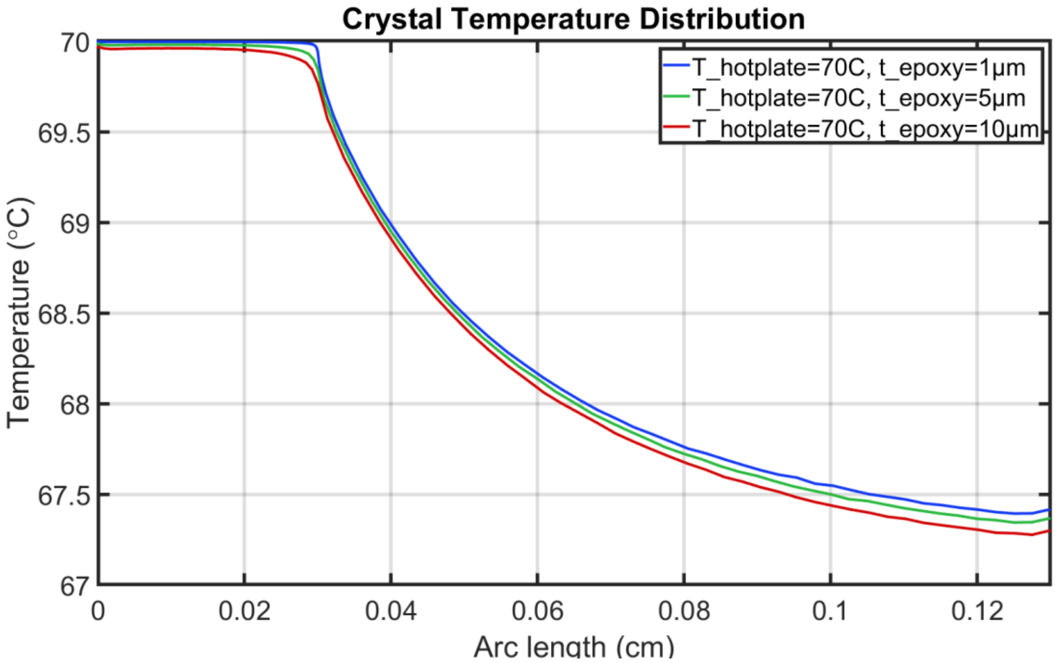

Supplementary Figure 29. FEM simulation of the effect of different layers of epoxy on TP MH particle at 70 °C

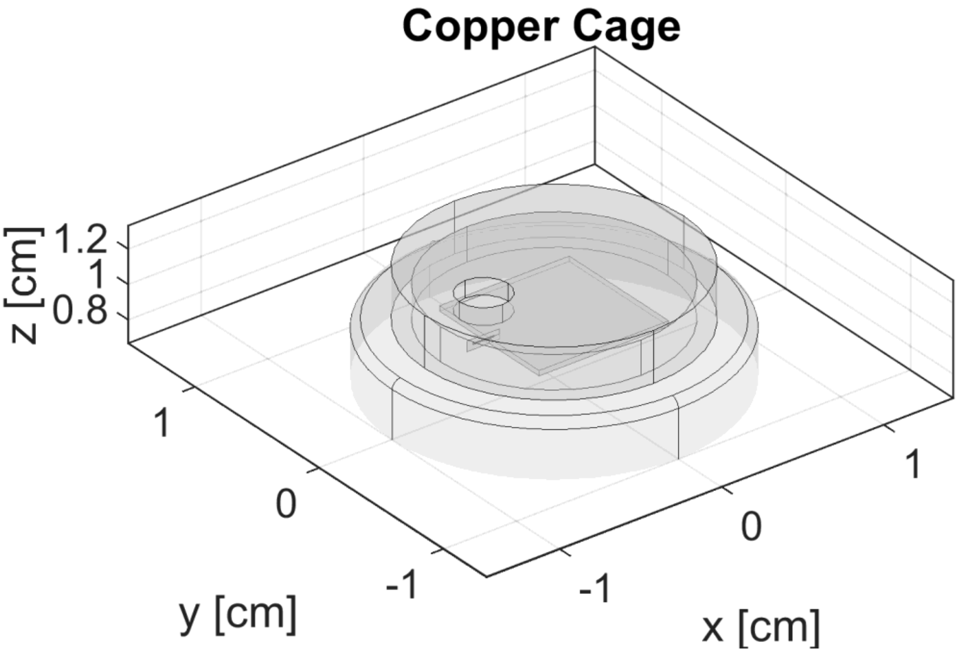

Supplementary Figure 30. Illustration of the copper cage for the PMTA using COMSOL

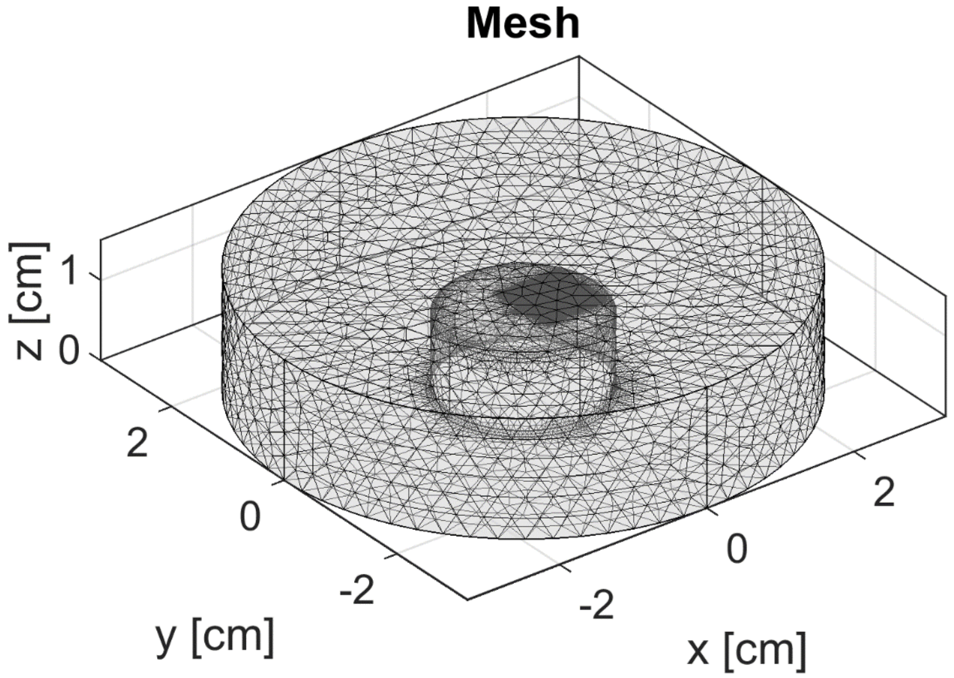

Supplementary Figure 31. **Illustration of the Linkam hot stage chamber.**

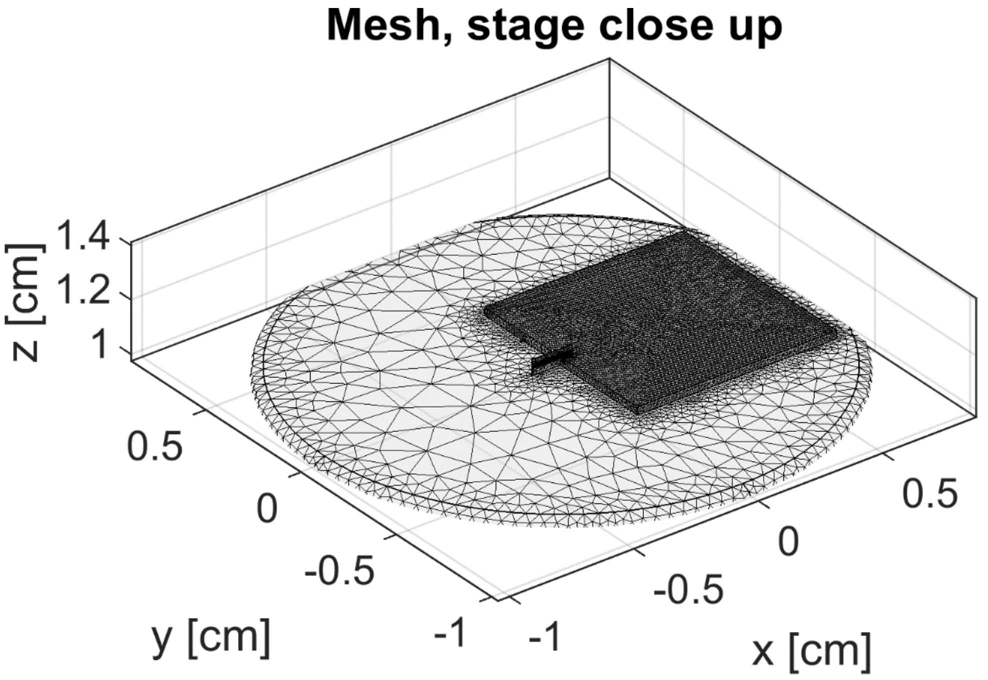

Supplementary Figure 32. Illustration of the aluminium block that was used to support the model particles.

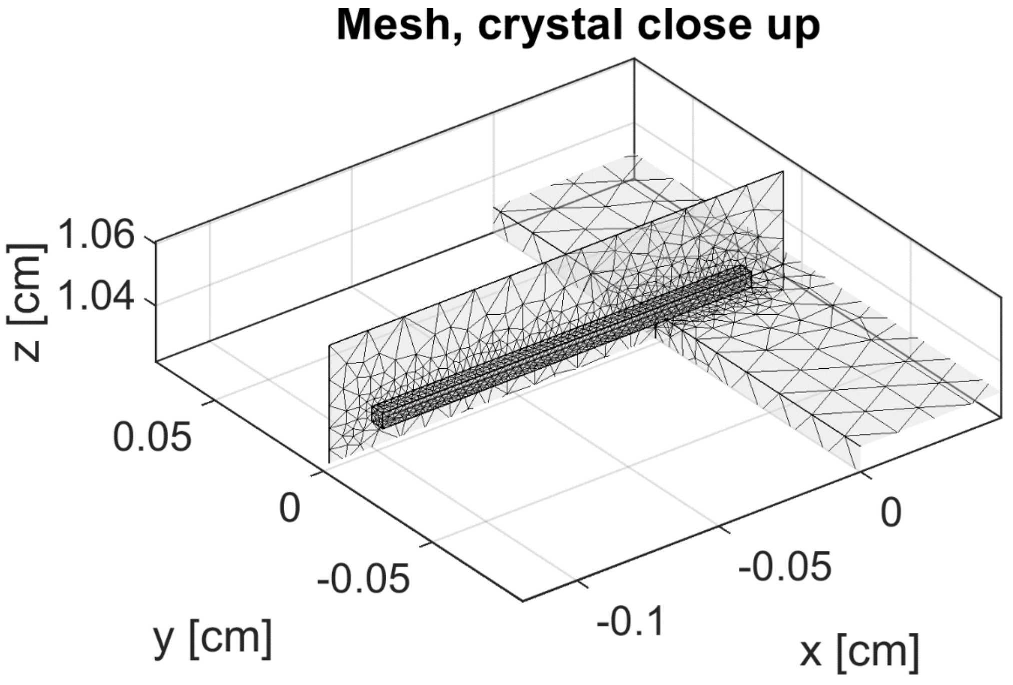

Supplementary Figure 33. Illustration of a TP MH particle suspended on an aluminium block.

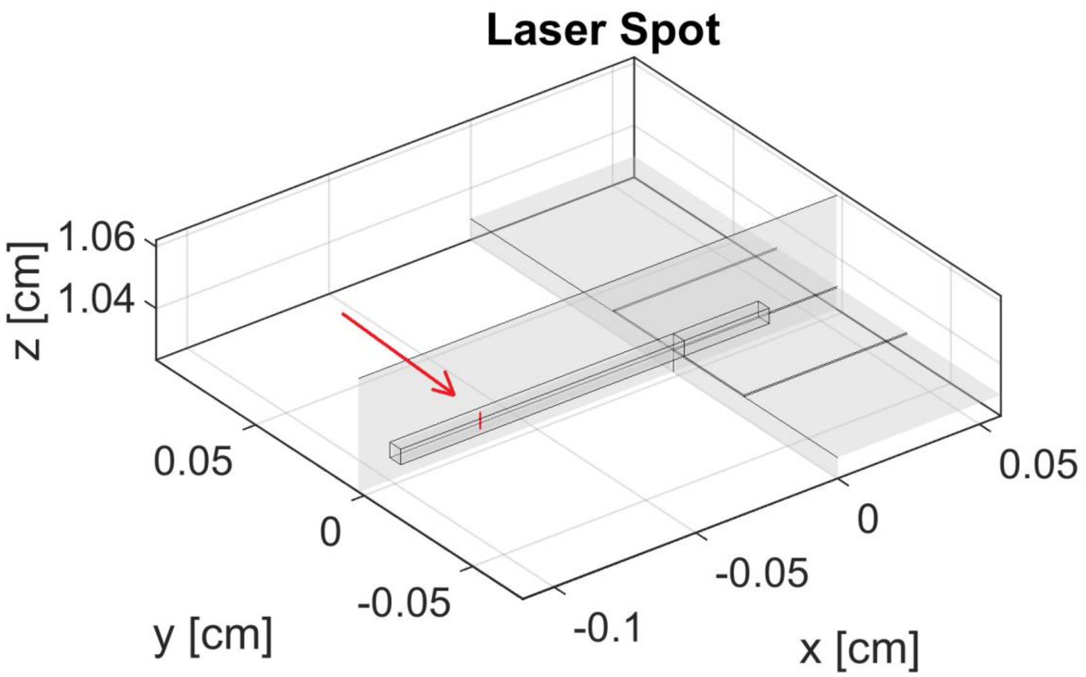

Supplementary Figure 34. Illustration of the laser spot from the LDV on a model particle using COMSOL.

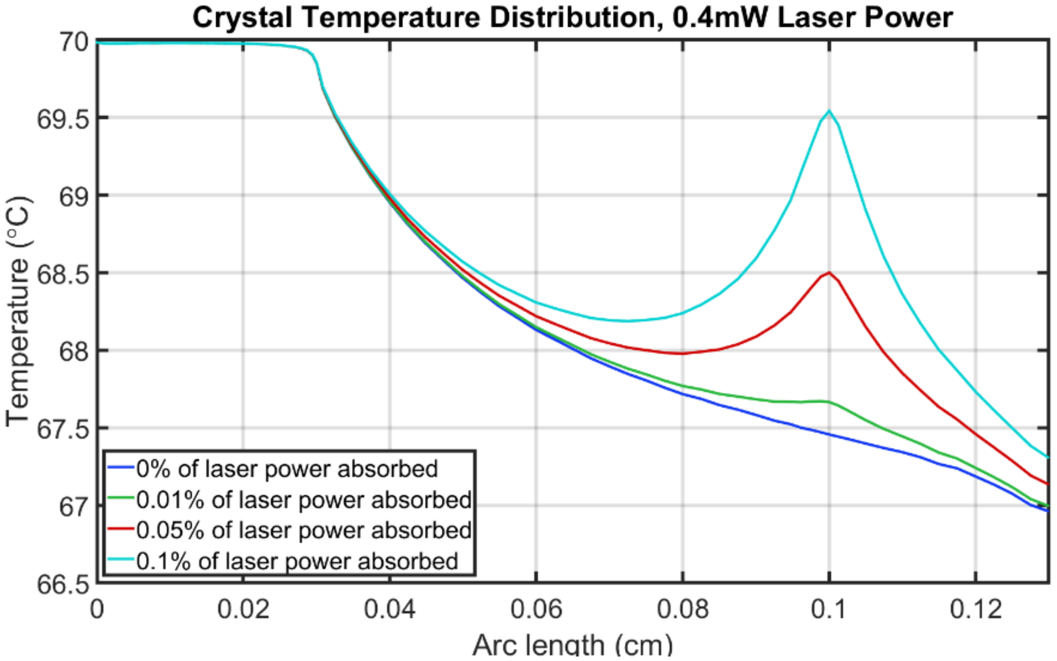

Supplementary Figure 35. Illustration of the laser power absorbed on a particle in varying laser powers. This is considering that the laser spot is stagnant on the particle and not moving as was done in the measurements presented in this work.

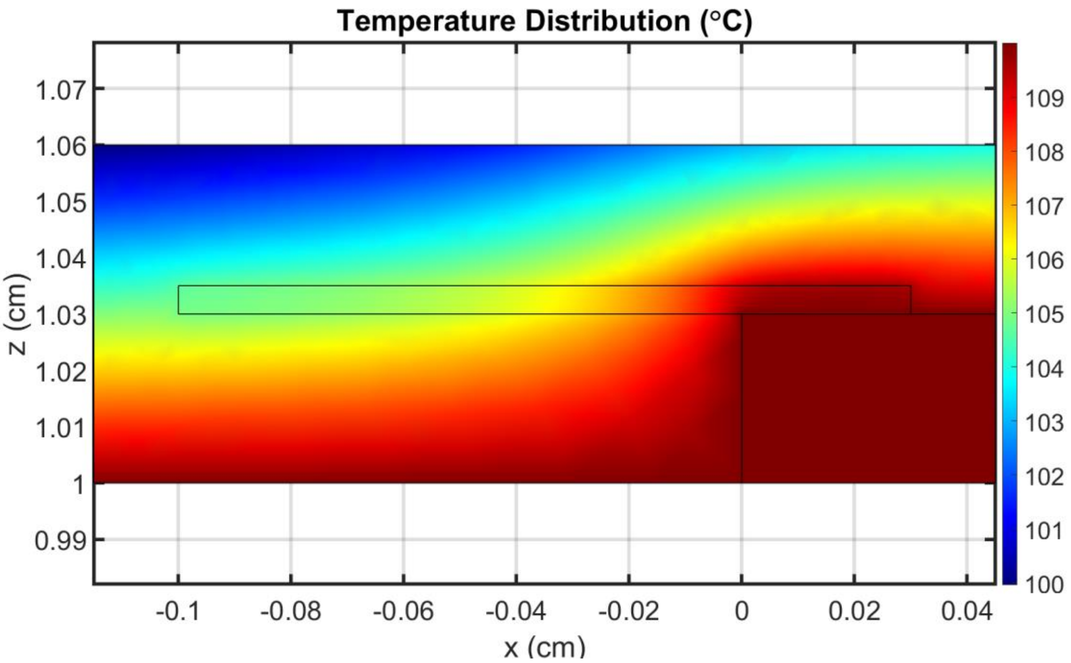

Supplementary Figure 36. FEM showing the temperature distribution on the Linkam hot plate with the crystal.

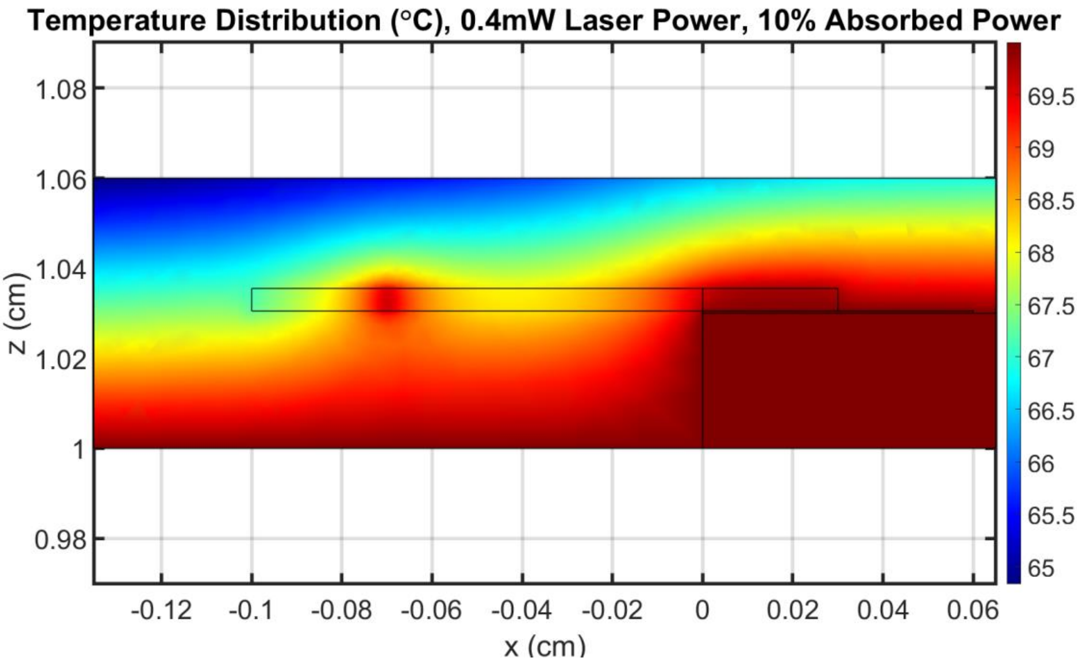

Supplementary Figure 37. FEM simulation of the effect of the laser power on the particle. This simulation is mimicing a situation where the laser spot remains stagnant on the particle whereas with the measurements done in this paper the laser spot was only present for a tenth of a second on a given spot on the particle.

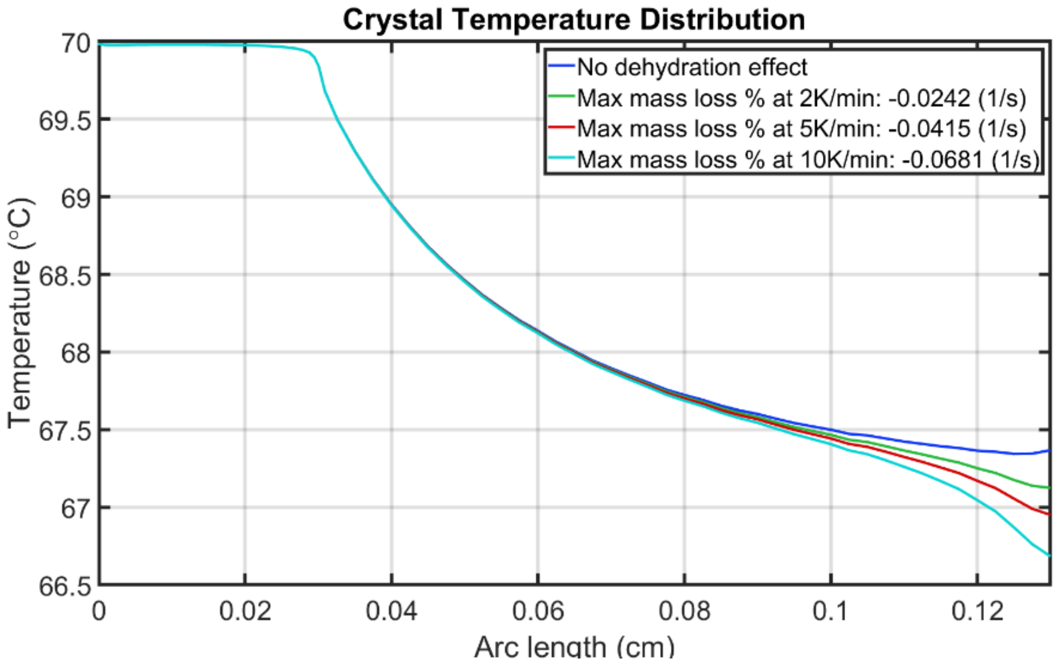

Supplementary Figure 38: FEM of the temperature profile along a TP MH particle during dehydration. The maximum mass loss was extracted from TGA data presented in the manuscript. This simulation is considering that there is no lid on the hot stage.

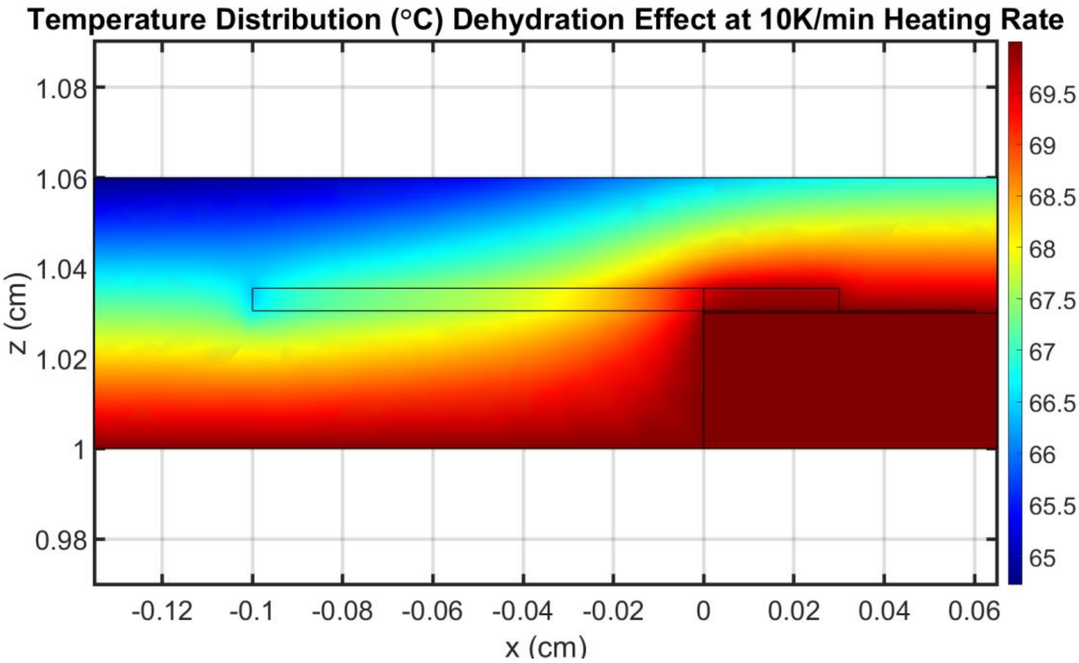

Supplementary Figure 39. FEM simulation of the effect of dehydration on the TP MH particle.

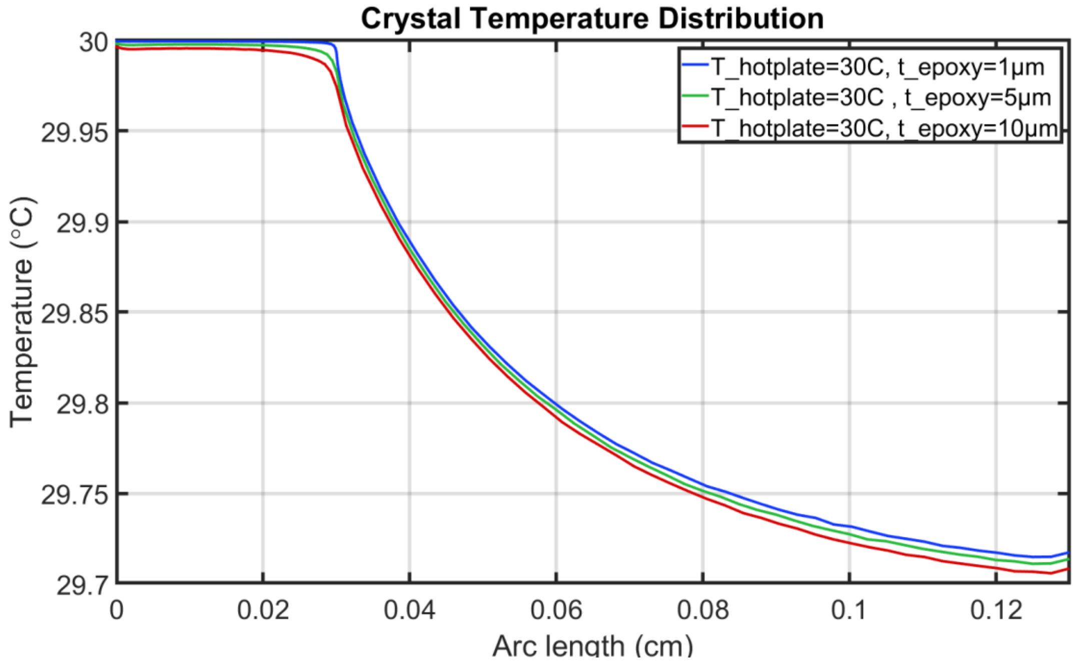

Supplementary Figure 40. FEM simulation of the effect of different layers of epoxy on TP MH particle at 30 °C.

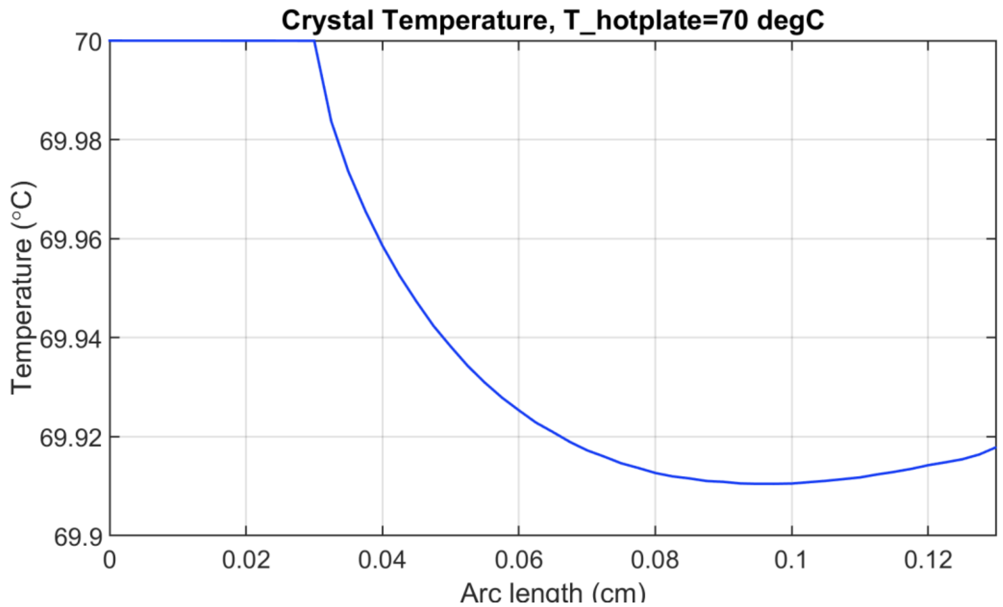

Supplementary Figure 41. FEM showing the temperature distribution of a TP MH cantilever at 70 °C.

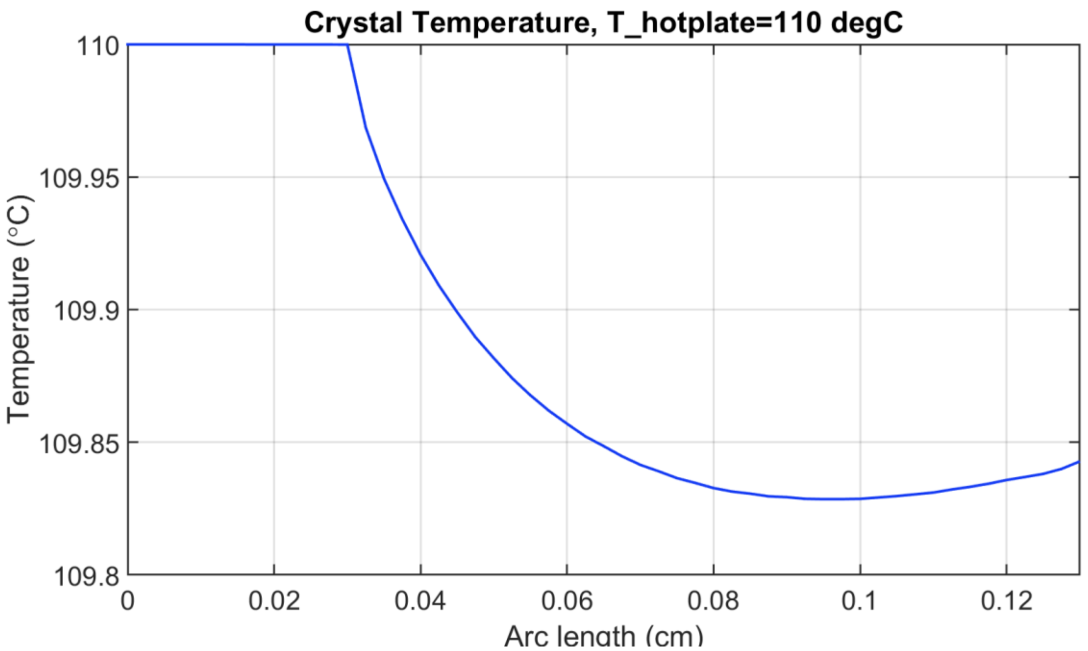

Supplementary Figure 42. Temperature distribution of a TP MH cantilever at 110 °C.

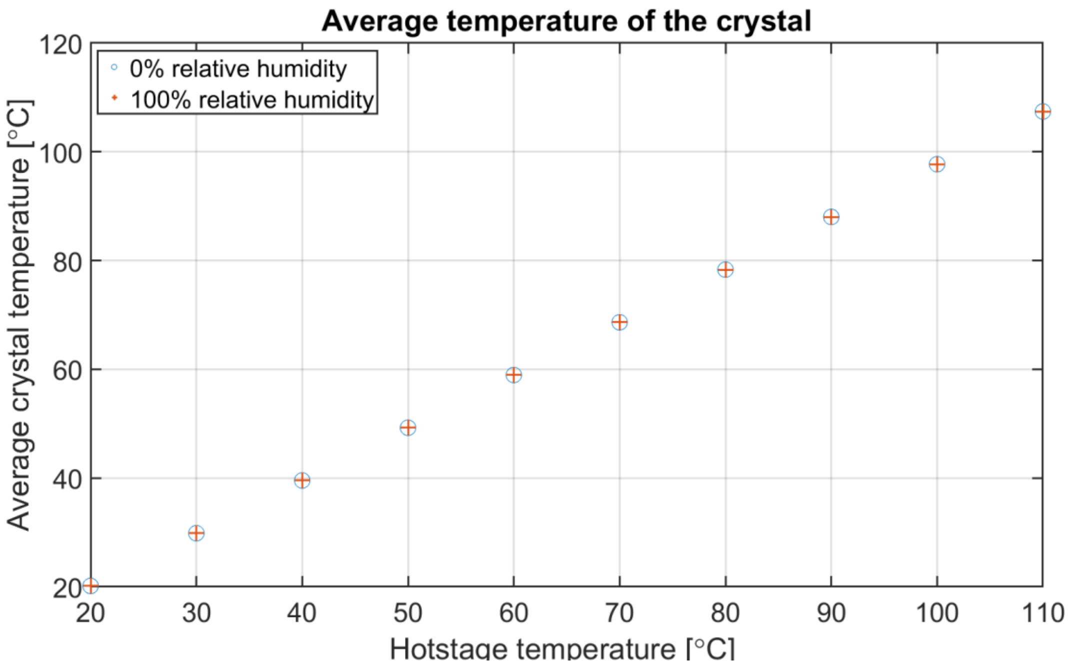

Supplementary Figure 43. Average temperature of the crystal at 0 and 100% RH.

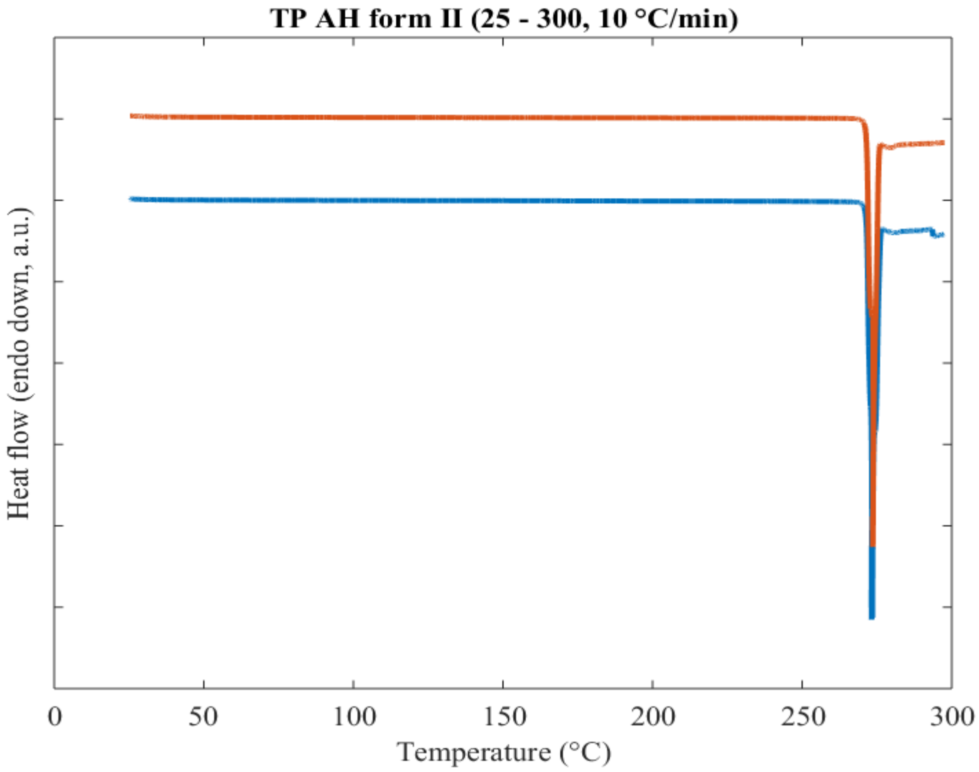

Supplementary Figure 44. DSC thermograms of TP AH form II (25-300 °C, 10 °C/min) that was used as the starting material for the recrystallization of TP MH.

Supplementary Figure 44 shows the DSC thermograms of TP AH form II. These thermograms confirmed the purity of the starting material that was used for the recrystallization experiments.

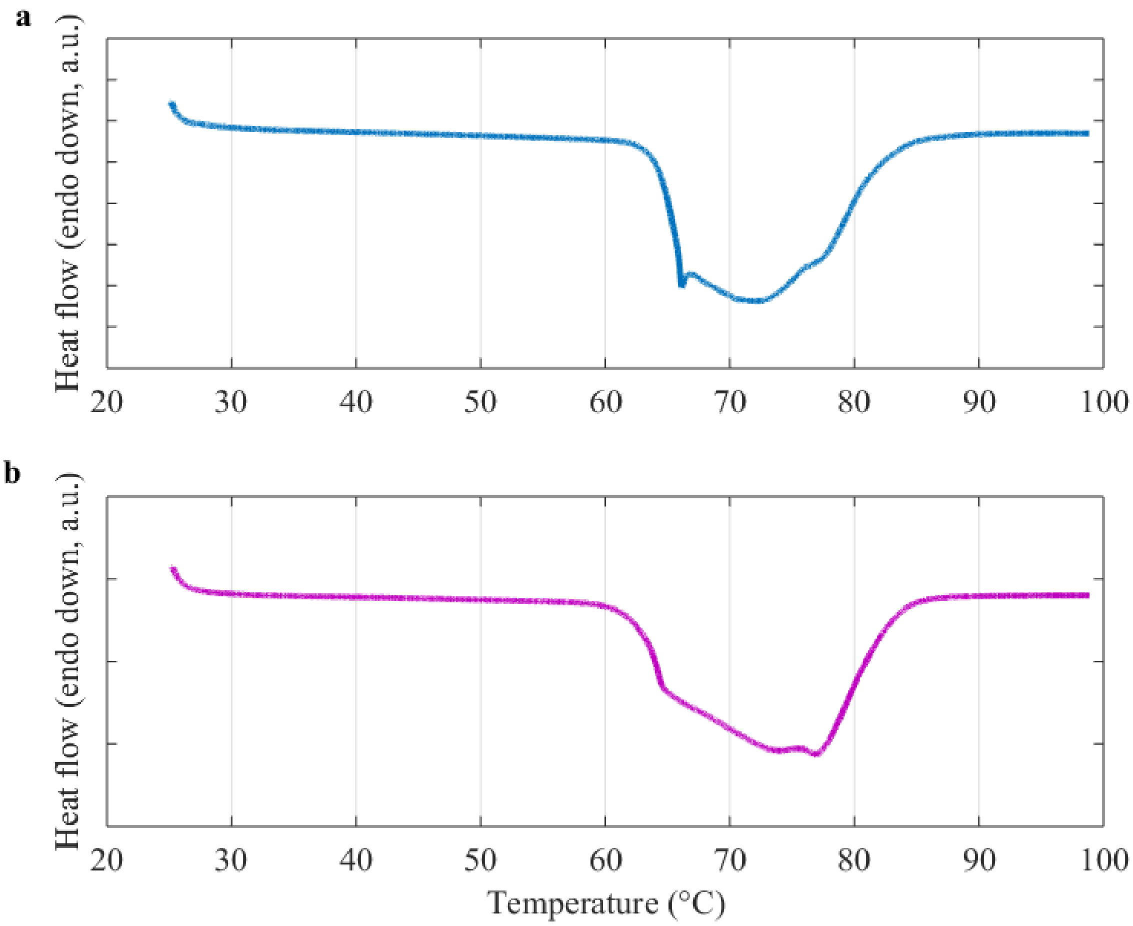

Supplementary Figure 45. DSC thermograms of TP MH from 25-100 °C, 5 °C/min.

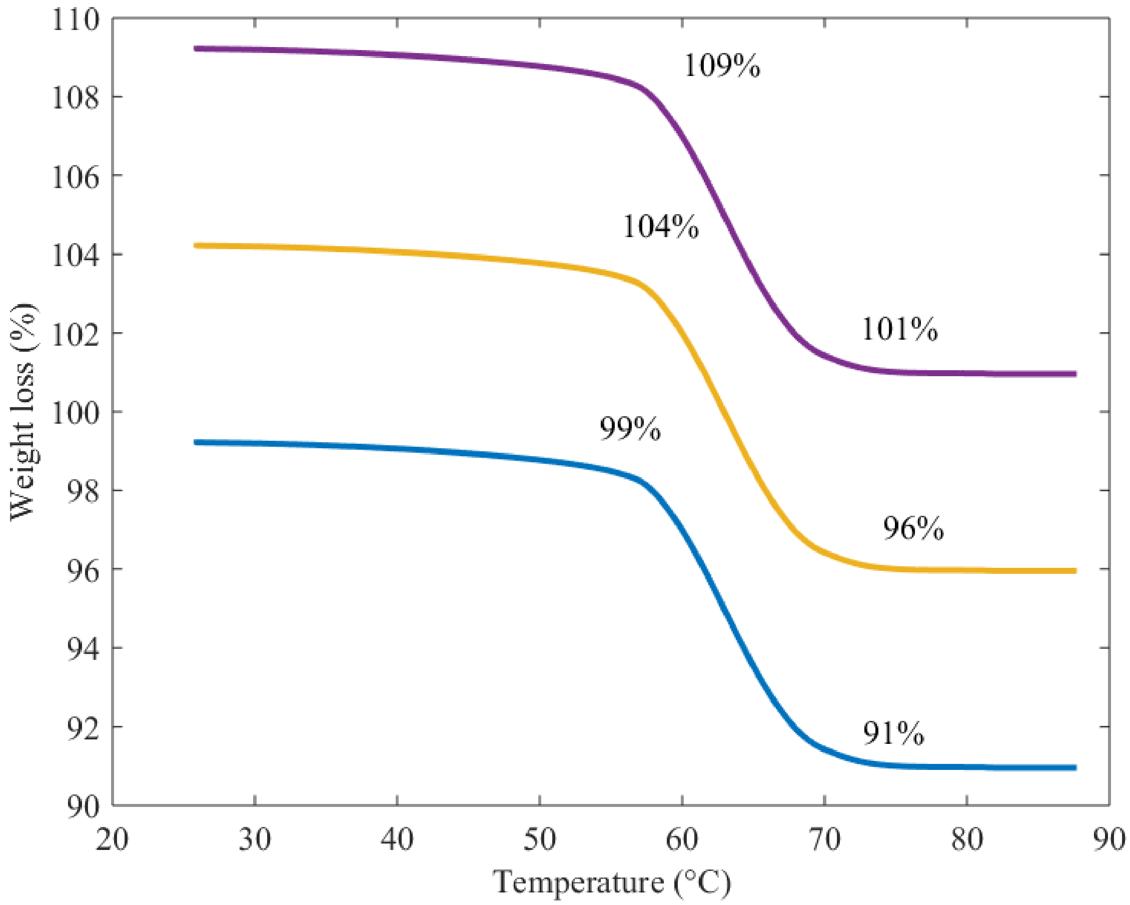

Supplementary Figure 46. TGA triplicates of TP MH at 25-90 °C, 5°C/min.

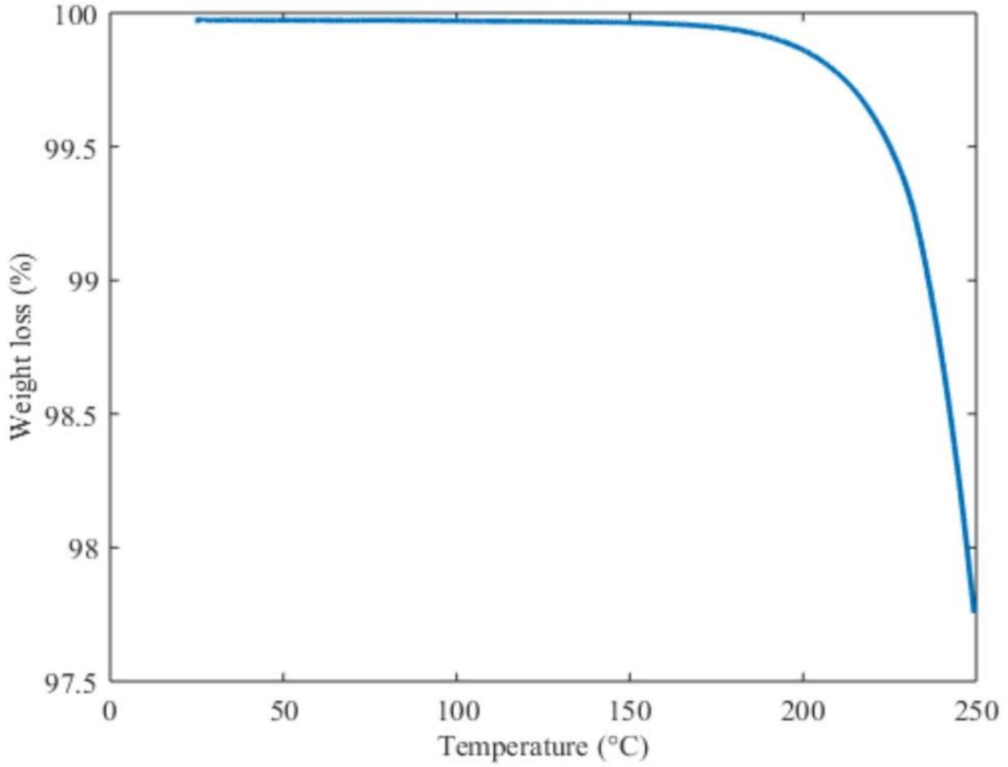

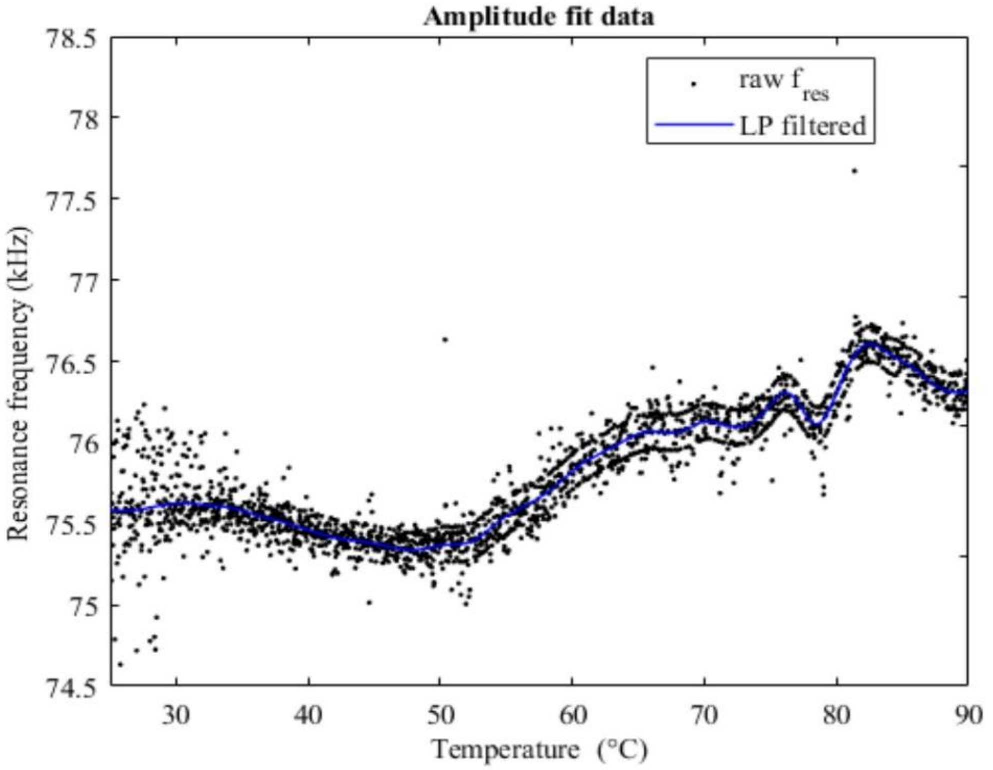

Supplementary Figure 47. TGA (25-250, 10°C/min) and PMTA thermograms of TP AH form II (25-90, 10°C/min) respectively.

Anhydrous theophylline was recrystallized as previously documented and a TGA run was performed in order to ensure that there was no water in the particles prior to PMTA analysis.

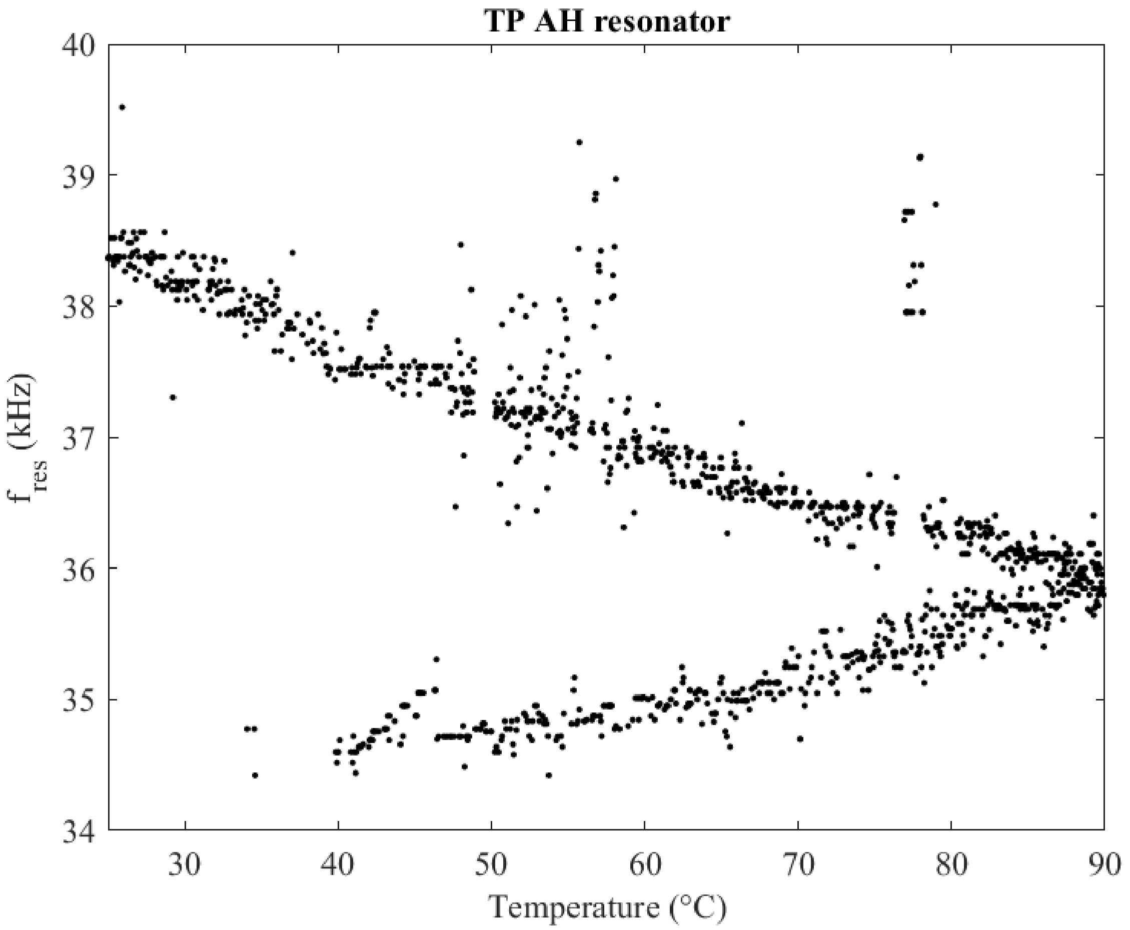

Supplementary Figure 48. Raw f_{res} data for a PMTA experiment using a TP AH particle (25-90-25, 5°C/min).

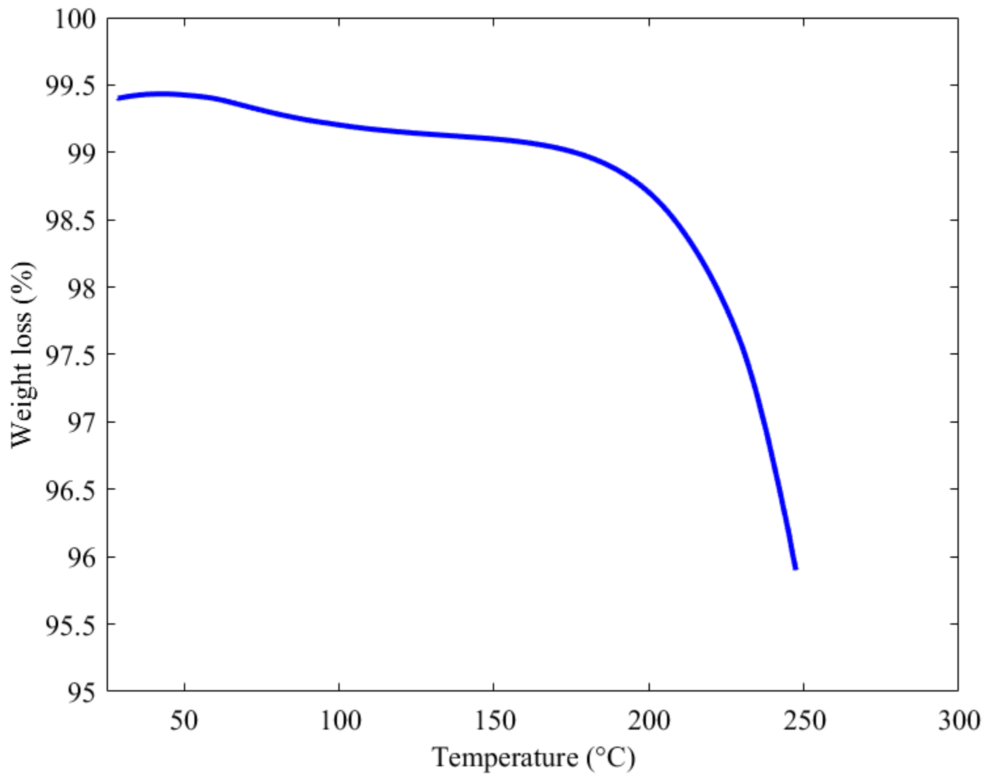

Supplementary Figure 49. TGA thermogram of dry collagen fibres (25-250 °C, 20 °C/min).

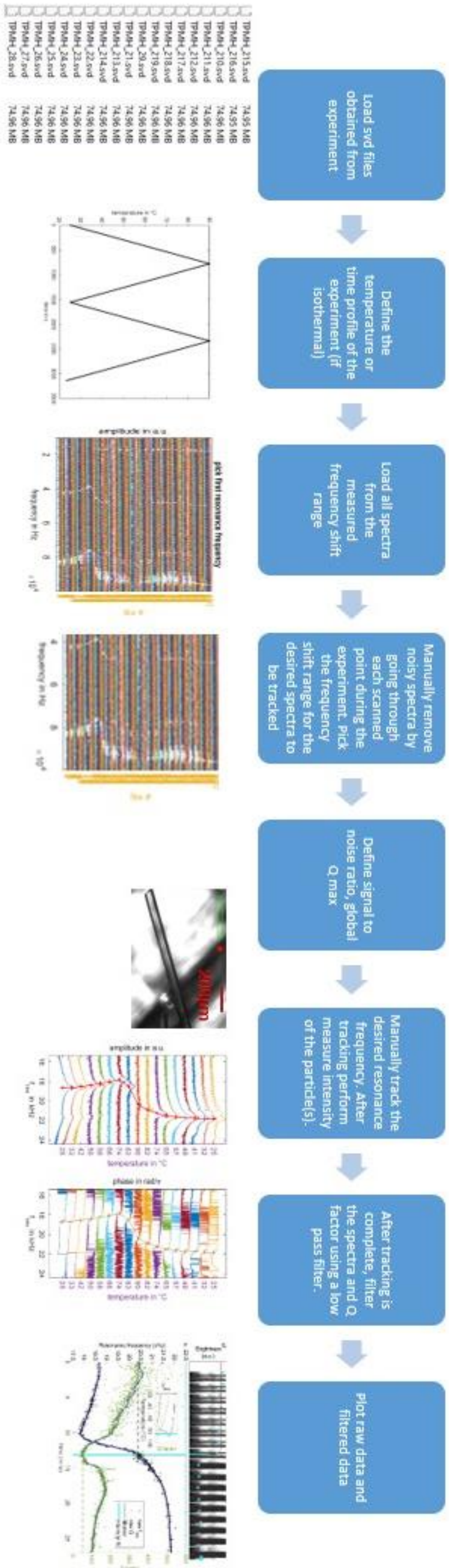

Supplementary Figure 50. An example of the data analysis steps that were used in order to plot the optical images, f_{res} and Q results from a single particle.

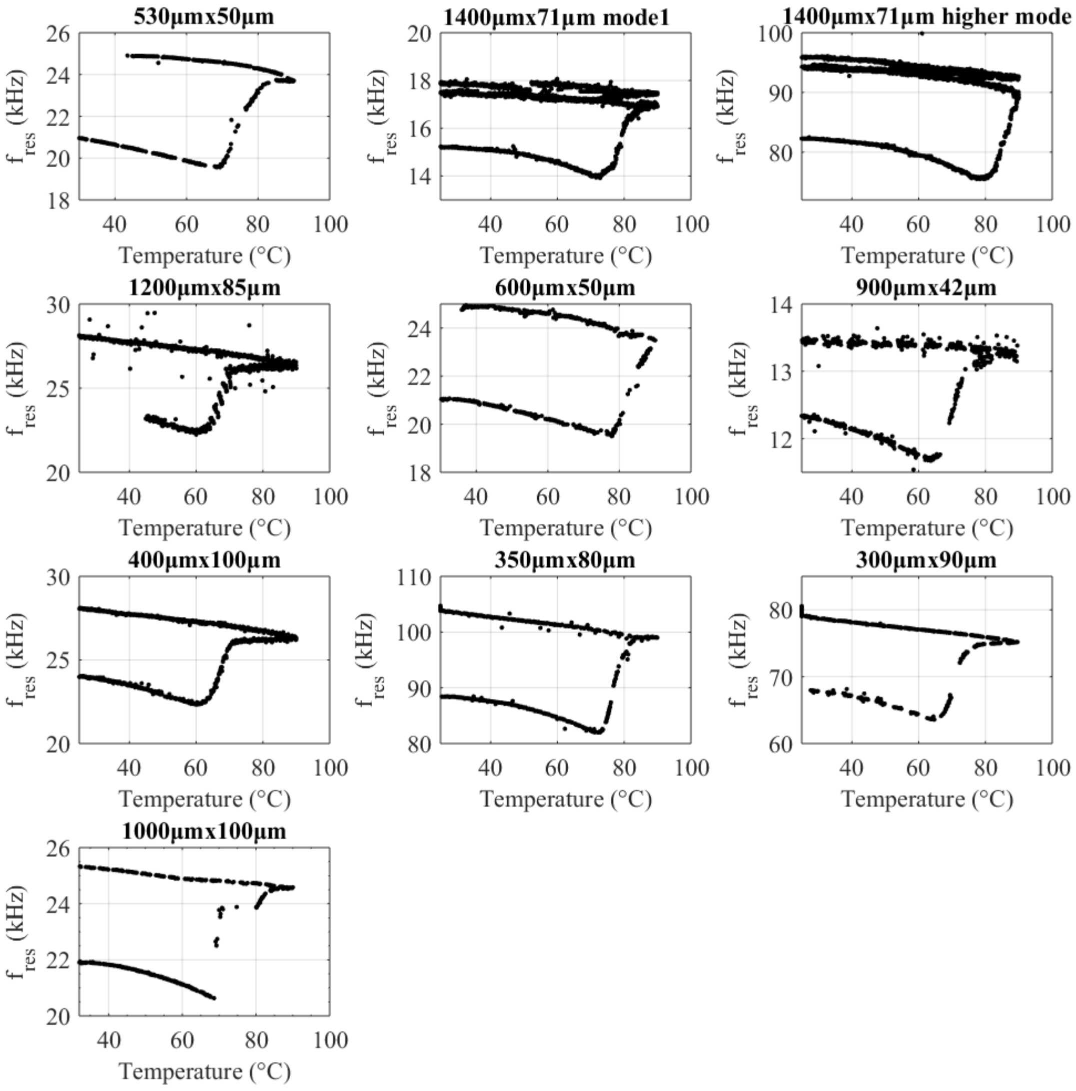

Supplementary Figure 51. Raw data of Figure 4 in main text.

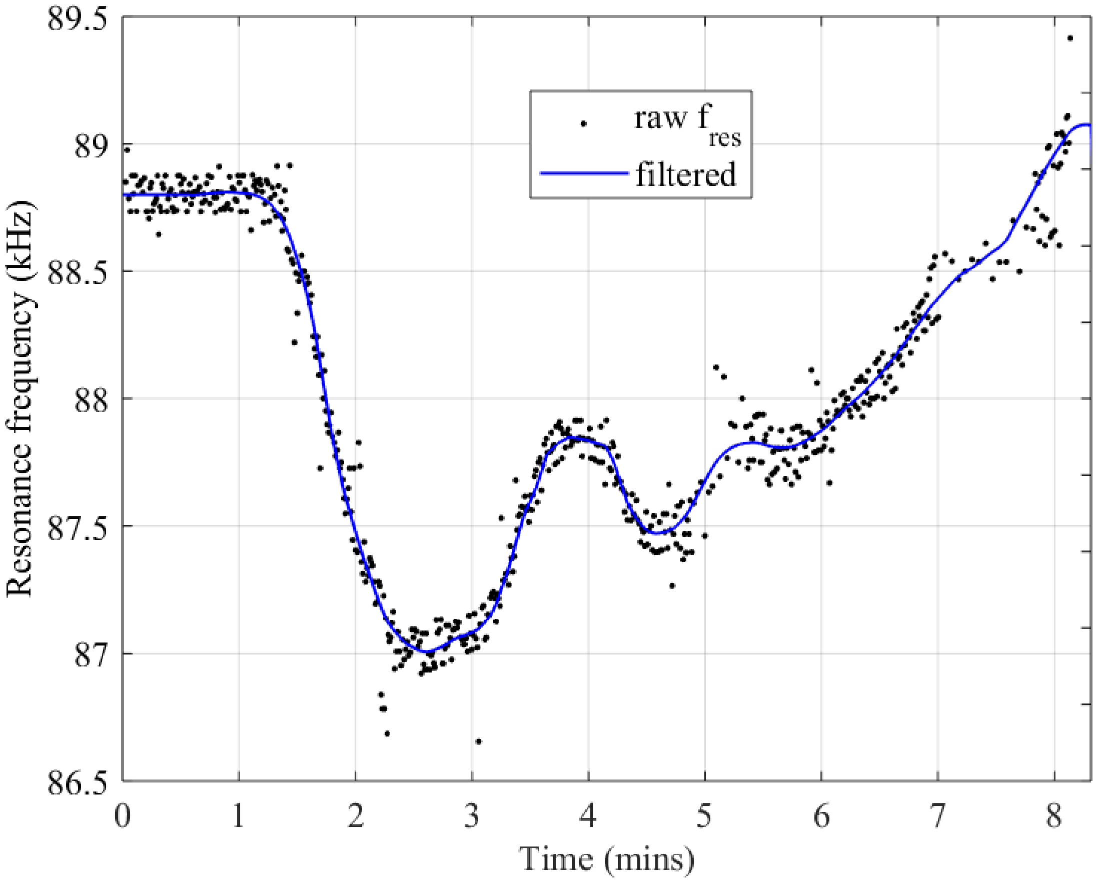

Supplementary Figure 52. Raw data of collagen PMTA experiment in Figure 6 of the main text
