# Supplementary material for: Single particles as resonators for thermomechanical analysis
Source: Nat Commun. 2020 Mar 6;11:1235. doi: 10.1038/s41467-020-15028-y (PMC7060253; doi:10.1038/s41467-020-15028-y)
Supplement: Supplementary file 3 — Description of Additional Supplementary Files [file 41467_2020_15028_MOESM3_ESM.docx]

**Description of Additional Supplementary Files**

File Name: Supplementary Video 1

Description: Dehydration of a small sized single TP MH particle as a cantilever (PMTA) at 25-90 °C, 5°C/min

File Name: Supplementary Video 2

Description: Dehydration of a single TP MH particle (approximately 1000µm in length) as a cantilever (PMTA) at 25-90 °C, 5°C/min

File Name: Supplementary Video 3

Description: Dehydration of a single TP MH particle (1000µm x 65µm) as a cantilever (PMTA) at 25-90 °C, 5°C/min

File Name: Supplementary Video 4

Description: Dehydration of a single TP MH particle as a cantilever (PMTA) at 50°C

File Name: Supplementary Video 5

Description: Dehydration of a single TP MH particle (530µm x 50µm) as a cantilever (PMTA) from 25-90°C, 5°C/min

File Name: Supplementary Video 6

Description: Dehydration of a “broken” single TP MH particle as a cantilever (PMTA) from 25-90°C, 5°C/min

File Name: Supplementary Video 7

Description: Visualisation of a vibrational mode of TP MH particle (1600μm x 66μm) cantilever from a Polytec vibrometer software (PSV 14.2).

File Name: Supplementary Video 8

Description: Visualisation of a vibrational mode of TP MH particle (1400μm x 71μm) cantilever from a Polytec vibrometer software (PSV 14.2).

File Name: Supplementary Video 9

Description: Visualisation of a vibrational mode for a TP MH particle cantilever that was obtained from a Polytec vibrometer software (PSV 14.2).

File Name: Supplementary Video 10

Description: Visualisation of a vibrational mode for a TP MH particle cantilever that was obtained from a Polytec vibrometer software (PSV 14.2).

File Name: Supplementary Video 11

Description: Visualisation of a vibrational mode for a TP MH particle cantilever that was obtained from a Polytec vibrometer software (PSV 14.2).

File Name: Supplementary Video 12

Description: Visualisation of a vibrational mode for a TP MH particle cantilever that was obtained from a Polytec vibrometer software (PSV 14.2).

File Name: Supplementary Video 13

Description: Visualisation of the first mode of vibration for a TP MH particle cantilever that was obtained from a Polytec vibrometer software (PSV 14.2).

File Name: Supplementary Video 14

Description: Visualisation of the first mode of vibration for a TP MH particle cantilever that was obtained from a Polytec vibrometer software (PSV 14.2).

File Name: Supplementary Video 15

Description: Visualisation of the second mode of vibration for a TP MH particle cantilever that was obtained from a Polytec vibrometer software (PSV 14.2).

File Name: Supplementary Video 16

Description: Visualisation of a higher mode for a TP MH particle cantilever that was obtained from a Polytec vibrometer software (PSV 14.2).

File Name: Supplementary Video 17

Description: Visualisation of the third mode of vibration for a TP MH particle cantilever that was obtained from a Polytec vibrometer software (PSV 14.2).

File Name: Supplementary Video 18

Description: Visualisation of the first mode of vibration for a collagen sample that was obtained from a Polytec vibrometer software (PSV 14.2).

File Name: Supplementary Video 19

Description: Thermomicroscopy of a collagen sample recoiling that was taken from the LDV

File Name: Supplementary Video 20

Description: Thermomicroscopy of a TP AH particle 25-90°C, 5°C/min undergoing a major geometric change

File Name: Supplementary Video 21

Description: Process of performing the tracking of the average TP MH particle intensity undergoing dehydration in MATLAB
